# Lighting the Way: Unveiling the Mechanisms of the Photoinduced Benzyl Alcohol Oxidation Using Tailored Bismuth‐Based Perovskite‐Inspired Microcrystals

**DOI:** 10.1002/cssc.202501555

**Published:** 2025-09-18

**Authors:** Daniele Conelli, Chiara Lo Porto, Anna Moliterni, Davide Altamura, Cinzia Giannini, Fabio Palumbo, Helena Mateos, Mokurala Krishnaiah, Tuhin Samanta, Kimmo Lahtonen, G. Krishnamurthy Grandhi, Paola Vivo, Gian Paolo Suranna, Roberto Grisorio

**Affiliations:** ^1^ Dipartimento di Ingegneria Civile, Ambientale del Territorio Edile e di Chimica (DICATECh) Politecnico di Bari Via Orabona 4 70125 Bari Italy; ^2^ CNR–Istituto di Cristallografia via G. Amendola, 122/O 70126 Bari Italy; ^3^ National Research Council c/o Department of Chemistry Institute of Nanotechnology (CNR‐NANOTEC) University of Bari ‘‘Aldo Moro’’ via Orabona 4 70125 Bari Italy; ^4^ Dipartimento di Chimica and CSGI (Center for Colloid and Surface Science) Università degli Studi di Bari ‘‘Aldo Moro’’ via Orabona 4 70125 Bari Italy; ^5^ Hybrid Solar Cells Faculty of Engineering and Natural Sciences Tampere University P.O. Box 541 FI‐33014 Tampere Finland; ^6^ Faculty of Engineering and Natural Sciences Tampere University P.O. Box 541 FI‐33014 Tampere Finland; ^7^ CNR‐NANOTEC – Institute of Nanotechnology c/o Campus Ecoteckne Via Monteroni 73100 Lecce Italy

**Keywords:** benzyl alcohol, oxidation, perovskite‐inspired materials, photocatalysis, reaction pathways

## Abstract

This study reveals that a fine balance between experimental conditions and catalyst design enhances the visible‐light‐driven oxidative process of benzyl alcohol in air. A comparative analysis of various bismuth‐based halide perovskite‐like materials with differing chemical compositions uncovers that the Cs_3_Bi_2_Br_9_ material outperforms the others, owing to its optimal bandgap and well‐aligned energy levels. Notably, small‐sized Cs_3_Bi_2_Br_9_ particles prepared under halide‐rich conditions promote the evolution of the benzaldehyde product with an unprecedented product evolution rate (14,525 μmol g^−1^ h^−1^), among the highest reported for heterogenous photocatalysis. The excess halide inhibits the particle growth and, being easily removed along with the bulky ammonium counterion by the washing steps after the synthesis, releases the metal catalytic sites at the surface responsible for the improved catalytic performances. Mechanistically, dehydrogenative (anyway leading to aldehydic products) and degradation pathways are found to be competitive with the substrate oxidation promoted by oxygen reactive species, while their individual contribution is correlated with the surface chemistry of the photocatalyst and is supported by theoretical calculations. Therefore, the insights of this study are considered fundamental for shining light on future chemical strategies for enriching the potential of perovskite materials toward sustainable transformations.

## Graphical abstract

This work illustrates how the introduction of surface halide vacancies in heterogenous Cs_3_Bi_2_Br_9_ microparticles facilitates the aerobic conversion of benzyl alcohol into the corresponding aldehyde. The strategy demonstrates broader applicability outperforming the conventional methods and underscoring surface defect potential in sustainable photocatalytic approaches.

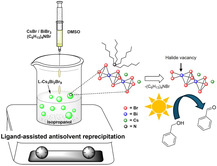

## 1 Introduction

The production of fine chemicals and highly valuable synthons from abundant and renewable resources, such as organic waste and biomass, has emerged as a critical research area aimed at developing circular economy strategies for a sustainable future.^[^ ^1^ ^]^ Benzyl alcohol (BA), which can be sustainably derived from lignin (one of the most abundant renewable feedstocks globally) is a particularly relevant starting material, as its selective oxidation to the corresponding aromatic aldehydes exemplifies a highly appreciated transformation with significant economic and industrial implications.^[^ ^2^ ^]^ Aromatic aldehydes, in particular, are widely used in various fields, including the industrial production of agrochemicals, pharmaceuticals, vitamins, dyestuff, drugs, fragrance, and additives. Developing more environmentally friendly and cost‐effective approaches in BA oxidation to replace conventional methods relying on stoichiometric oxidants or precious metal catalysts is then imperative. Among emerging sustainable approaches, the design of catalytic methods activated by solar energy utilizing the advantages of nanostructured materials, although still in early development, represent a promising solution to address these critical challenges.

Photocatalysis combines the exciton splitting aptitude of inorganic semiconductors with the activity of the catalytic sites exploiting charges concentration at the photocatalyst (PC) surface to promote redox processes with selected substrates.^[^ ^3^ ^]^ Among emerging PCs, metal halide perovskites (MHPs) have gained substantial attention due to their tunable optoelectronic properties and efficient charge separation, enhancing interactions between PCs and substrates, thus significantly improving photocatalytic efficiency.^[^ ^4^, ^5^ ^]^ Among these materials, bismuth‐based perovskites—such as Cs_3_Bi_2_X_9_ (X = Cl, Br or I)—stand out as promising alternatives to lead‐based perovskites due to their lower toxicity, wider bandgaps at the same halide composition, and improved stability.^[^ ^6^ ^]^ While product evolution was demonstrated for Pb‐based perovskite materials with product evolution rates not exceeding the value of 5200 μmol g^−1^ h^−1^,^[^ ^7^ ^]^ recent studies have explored the potential of nanometric Cs_3_Bi_2_X_9_ quantum dots in BA oxidation reactions, demonstrating improved charge separation and light absorption through bandgap modulation.^[^ ^8^ ^]^ Recently, BA was utilized in the formation of the anhydrides^[^ ^9^ ^]^ or amides^[^ ^10^ ^]^ under the proper reaction condition and partners. High oxidation rates (up to 10,200 μmol g^−1^ h^−1^) are achieved over perovskite/TiO_2_ composites^[^ ^11^, ^12^ ^]^ or using carbon nitride as the support,^[^ ^13^ ^]^ which are superior to that of unsupported materials with inevitable implications on manufacturing costs of the PC. Notwithstanding the synthesis method could play a key role in dictating the relevant PC performance through influencing the surface area containing the active sites,^[^ ^14^ ^]^ no studies directly compare the photocatalytic activities of MHPs with different (surface) compositions and morphologies in the same transformation. Addressing this research gap could provide critical insights, enabling more targeted and efficient design strategies for high‐performance perovskite PCs.

In this study, we investigate the impact of catalyst composition on the kinetics and selectivity of the prototypical photoinduced oxidation of BA in air to produce benzaldehyde (BAD). We confirm that the chosen synthetic strategy provides crucial control over the surface area (via particle dimensions), surface composition, and energy levels of the corresponding materials. We introduce the ligand‐assisted antisolvent reprecipitation (LARP) strategy to synthesize a heterogeneous Cs_3_Bi_2_Br_9_ PC aiming at an unprecedented control over its surface chemistry. Compared to conventionally prepared Cs_3_Bi_2_Br_9_ sample, which exhibited a product evolution rate of 2500 μmol g^−1^ h^−1^ in air, the LARP‐derived Cs_3_Bi_2_Br_9_ (L‐Cs_3_Bi_2_Br_9_) material achieved an exceptional product evolution rate of 14,525 μmol g^−1^ h^−1^. For the first time, the contribution of the three competitive reaction pathways (aerobic oxidation, anaerobic dehydrogenative oxidation, and degradation) was rationalized based on the PC's surface chemistry and reaction conditions. We are confident that the remarkable results achieved with the PCs designed for this study could pave the way for the rational design of advanced low‐toxicity perovskite‐based materials for sustainable organic transformations, consolidating their potential for large‐scale applications within a solar‐driven circular economy.

## 2 Results and Discussion

**Figure 1:**
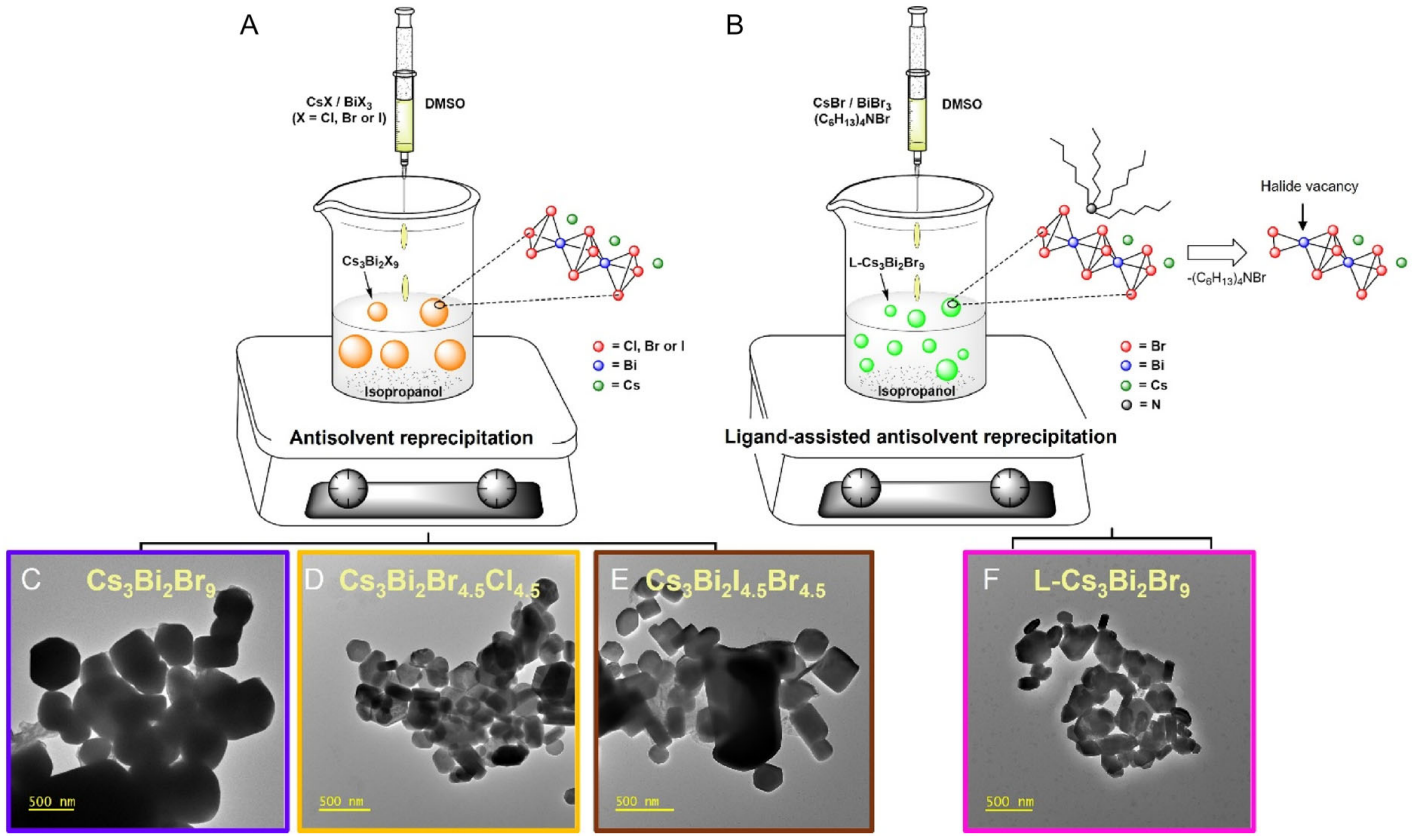
A) Schematic representation of the synthesis of Cs_3_Bi_2_X_9_ (X = Cl, Br, I) via antisolvent precipitation from a DMSO solution of cesium and bismuth halides in isopropanol. B) Schematic representation of the synthesis of L‐Cs_3_Bi_2_Br_9_ in the presence of THAB, added to modulate particle size and surface properties. C–F) TEM images of Cs_3_Bi_2_Br_9_, Cs_3_Bi_2_Cl_4.5_Br_4.5_, Cs_3_Bi_2_I_4.5_Br_4.5_, and L‐Cs_3_Bi_2_Br_9_.

Conventional Cs_3_Bi_2_X_9_ (X = Cl, Br, or I) samples were synthesized via antisolvent reprecipitation by combining cesium halides and bismuth halides in a 3:2 molar ratios in dimethyl sulfoxide (DMSO) followed by a rapid injection in isopropanol, which promoted the immediate crystallization of the bismuth perovskite‐like materials (**Figure** **1**A). The solids were collected by centrifugation, washed with solvent, and dried under vacuum. The materials prepared following this approach are Cs_3_Bi_2_Br_9_ and the corresponding mixed‐halide analogs (Cs_3_Bi_2_I_4.5_Br_4.5_ and Cs_3_Bi_2_Br_4.5_Cl_4.5_) with a formal equimolar ratio between the halides. Aiming at enabling a control over particle size and surface properties of the corresponding Cs_3_Bi_2_Br_9_ sample, we introduced tetrahexylammonium bromide (THAB:BiBr_3_ = 1:2 molar ratio) during the synthesis (Figure 1B). Competing with cesium cations, it is expected that the large and labile organic ammonium cations inhibit the particle growth (leading to small‐size particles in comparison with the conventional approach) and generate a different surface elemental composition (introducing halide vacancies).^[^ ^15^ ^]^

The internal and surface composition of the synthesized PCs were initially approached by using thermogravimetric analysis (TGA) under a nitrogen atmosphere. Conventionally prepared perovskites (Cs_3_Bi_2_Br_9_, Cs_3_Bi_2_Cl_4.5_Br_4.5_, and Cs_3_Bi_2_I_4.5_Br_4.5_) revealed two distinct thermal events for each material, ascribable to sublimation of the two components of the perovskite structure (CsX and BiX_3_) after decomposition (Figure 1SA, Supporting Information). An additional event at ≈150 °C was observed in the case of Cs_3_Bi_2_I_4.5_Br_4.5_, which can be attributed to the loss of the residual solvent molecules (DMSO or *iso*propanol). This behavior is consistent with previous studies on perovskite materials.^[^ ^16^, ^17^ ^]^ It is reasonable to suppose that the layered structure of the iodine‐containing material can accommodate the crystallization solvents more prominently compared to the other halide compositions. As reported in Figure 1SB, Supporting Information, the TGA plot of L‐Cs_3_Bi_2_Br_9_ reveals the absence of thermal transitions attributable to an organic component (THAB) and an increased decomposition temperature (536 °C vs. 518 °C), suggesting enhanced thermal robustness probably due to the regular composition of the material surface.^[^ ^18^, ^19^ ^]^ This experiment confirms the “transitory” role of the organic ligand (THBA) in the preparation of L‐Cs_3_Bi_2_Br_9_, because intervening during the particle growth but removed after the washing step, de facto modifying only the surface composition of the resulting material (vide infra).

**Figure 2:**
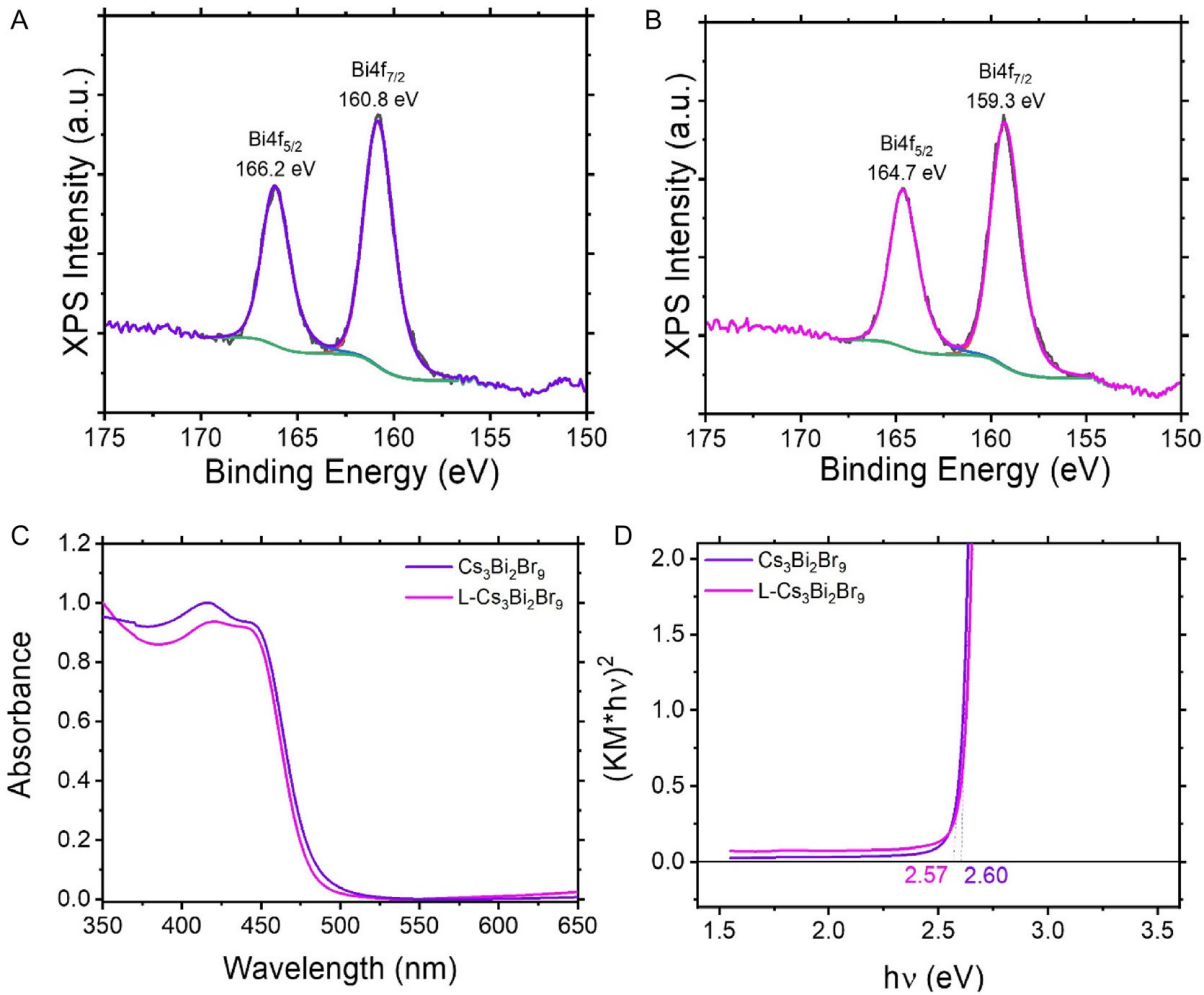
A) Core level high resolution XPS spectrum of Bi in conventionally prepared Cs_3_Bi_2_Br_9_ microcrystals. B) Core level high resolution XPS spectrum of Bi in L‐Cs_3_Bi_2_Br_9_ microcrystals. C) Diffuse reflectance spectra of conventionally prepared Cs_3_Bi_2_Br_9_ and THAB‐modified Cs_3_Bi_2_Br_9_ (L‐Cs_3_Bi_2_Br_9_) materials. D) Kubelka–Munk transformation of the relevant absorption spectra, showing a slight reduction in band gap for L‐Cs_3_Bi_2_Br_9_.

**Figure 3:**
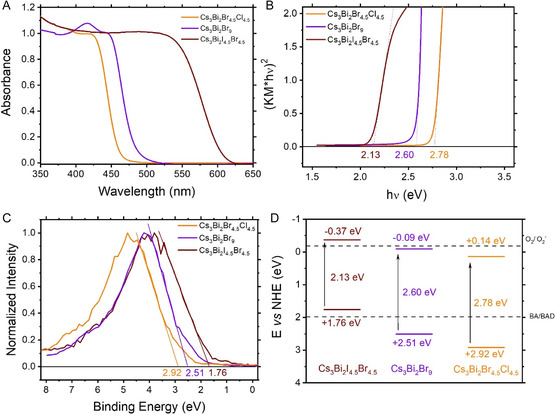
A) Diffuse reflectance spectra of Cs_3_Bi_2_Br_9_, Cs_3_Bi_2_Cl_4.5_Br_4.5_, and Cs_3_Bi_2_I_4.5_Br_4.5_, showing absorption edge shifts upon halide substitution. B) Kubelka–Munk transformed absorption spectra used for bandgap determination. C) Valence band maximum (VBM) positions extracted from XPS. D) Energy level diagram illustrating the electronic structure of Cs_3_Bi_2_X_9_ perovskites.

**Figure 4:**
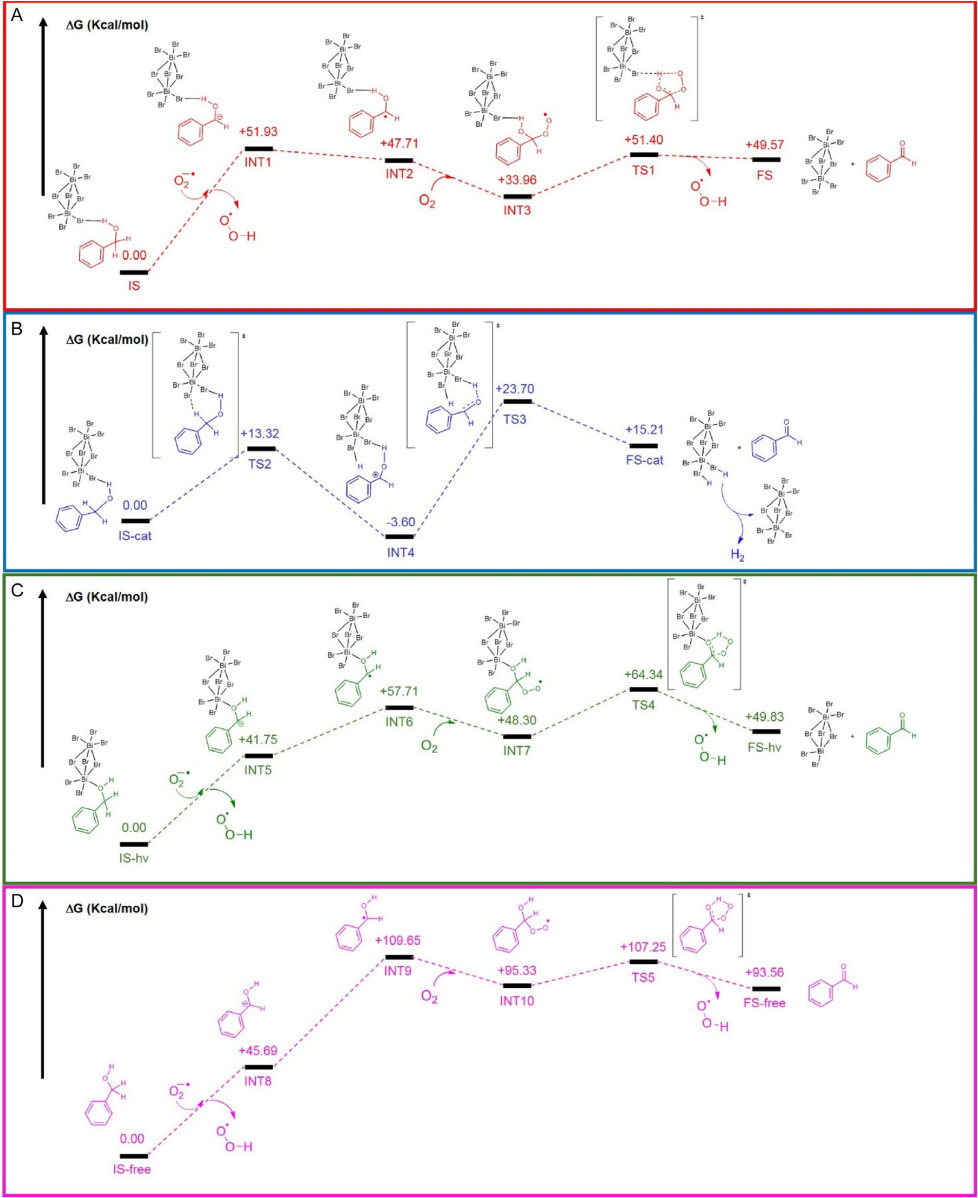
Computed reaction energy profiles for the oxidation of BA under various conditions. A) Aerobic pathway catalyzed by the [Bi_2_Br_9_]^3−^ cluster. B) Anaerobic pathway catalyzed by the [Bi_2_Br_9_]^3−^ cluster. C) Aerobic pathway catalyzed by the halide‐deficient [Bi_2_Br_8_]^2−^ cluster. D) Aerobic pathway in the absence of a catalytic surface (noncatalyzed reference). All relative energies are reported in kcal mol^−1^ and correspond to gas‐phase optimized geometries, with single‐point energy corrections computed in ACN using the CPCM solvation model. Intermediates and transition states are labeled accordingly to facilitate comparison across different mechanistic scenarios.

The morphology and elemental composition of the synthesized materials were investigated using transmission electron microscopy (TEM) and scanning electron microscopy (SEM) coupled with energy‐dispersive X‐ray spectroscopy (EDX). The TEM image of Cs_3_Bi_2_Br_9_ (Figure 1C) suggests the formation of prismoidal particles (≈500 nm of edge). EDX analysis of these particles revealed a homogeneous distribution of the elements (Figure 2S, Supporting Information), confirming their successful incorporation in the perovskite structure. The experimental composition (Cs_4.8_Bi_2.0_Br_9.0_) closely resembles the theoretical ratio between bismuth and bromine, while showing an excess cesium, probably due to a preferential allocation of this element at the particle surfaces.^[^ ^20^, ^21^ ^]^ Analogously, the TEM image of Cs_3_Bi_2_Cl_4.5_Br_4.5_ (Figure 1D) shows smaller prismoidal particles. While the EDX mapping confirmed the homogeneous distribution of the four constituting elements, their atomic composition—Cs_4.8_Bi_2.0_Br_5.4_Cl_4.6_—deviates from the ideal stoichiometry of Cs_3.0_Bi_2.0_Br_4.5_Cl_4.5_, indicating a slight excess of halides and cesium (Figure 3S, Supporting Information).^[^ ^22^ ^]^ Also, the morphology of Cs_3_Bi_2_I_4.5_Br_4.5_ (Figure 1E) revealed particles smaller in size, if compared to those of the conventionally prepared Cs_3_Bi_2_Br_9_. This reduction in particle size can be attributed to the influence of iodine (or chlorine), which likely inhibits crystal growth, resulting in smaller particles with a higher surface area. EDX analysis of this material confirmed the homogeneous distribution of Cs, Bi, Br, and I, supporting the incorporation of iodine into the perovskite structure (Figure 4S, Supporting Information). The elemental composition obtained from EDX (Cs_4.6_Bi_2.0_I_5.9_Br_4.4_) is consistent with the expected incorporation of iodine and confirms the tendency toward an excess halide in the materials prepared by a conventional reprecipitation method. This finding agrees with previous reports indicating that iodine substitutions can effectively control the size and morphology of perovskite materials.^[^ ^19^ ^]^ TEM analyses on L‐Cs_3_Bi_2_Br_9_ evidenced smaller prismoidal particles (≈200 nm of edge) with plausibly increased surface area (Figure 1F). EDX mapping (Figure 5S, Supporting Information) underscores that this sample proposes an experimental stoichiometry (Cs_4.3_Bi_2.0_Br_8.6_) quite different from its conventionally prepared counterpart (Cs_4.8_Bi_2.0_Br_9.0_). In particular, the reduction of the cesium amount in the L‐Cs_3_Bi_2_Br_9_ sample suggests competitive action between Cs^+^ and the organic ammonium cation during crystallization.^[^ ^23^, ^24^, ^25^ ^]^ At the same time, the halide deficiency is the result of the organic component removal, as depicted in Figure 1B.

To validate the morphological deductions on the particle sizes, we performed dynamic light scattering (DLS) measurements. As reported in Figure 6S, Supporting Information, the average particle sizes of Cs_3_Bi_2_Br_9_, Cs_3_Bi_2_Cl_4.5_Br_4.5_, and Cs_3_Bi_2_I_4.5_Br_4.5_ were found to be 2.89 ± 0.52, 4.56 ± 0.50, and 2.03 ± 0.36 μm, respectively, indicating that the iodide‐containing material exhibits smaller particles in analogy with qualitative TEM observations. At the same time, DLS analysis on L‐Cs_3_Bi_2_Br_9_ showed that its average particle size (2.09 ± 0.49 μm) is effectively influenced by the THAB presence inhibiting the growth with respect to the conventional approach (Figure 6S, Supporting Information).

To gain insight into the surface properties of the synthesized particles, we carried out Zeta potential measurements and the obtained values on the materials prepared by the conventional method are found to be +4.11, −7.12, −21.18, and −20.81 mV for Cs_3_Bi_2_Br_9_, Cs_3_Bi_2_Cl_4.5_Br_4.5_, Cs_3_Bi_2_I_4.5_Br_4.5_, and L‐Cs_3_Bi_2_Br_9_, respectively (Figure 7S, Supporting Information). This result indicates that the surface charge of conventionally prepared materials can be influenced by the halide composition, while, with the same element composition, by the synthesis method. The more negative surface charge suggests limited interparticle interactions due to repulsion^[^ ^26^ ^]^ and consequently increasing exposed catalytic sites density. These properties are crucial for optimizing charge transfer at the solid‐liquid interface, a key factor in heterogeneous photocatalysis.^[^ ^27^ ^]^

X‐ray photoelectron spectroscopy (XPS) analyses were carried out to investigate the valence states, chemical environments, and surface compositions of constituent elements in conventional Cs_3_Bi_2_Br_9_, ligand‐modified L‐Cs_3_Bi_2_Br_9_, and mixed‐halide Cs_3_Bi_2_X_9_ (X = Cl, Br, I) samples. A comparative analysis of the two all‐bromide samples revealed two characteristic peaks in the Cs 3*d* core‐level spectrum (Figure 8S, Supporting Information), located at binding energies of 738.3 and 724.5 eV, corresponding to the Cs 3*d*_3/2_ and Cs 3*d*_5/2_ spin–orbit components, respectively. These values are in agreement with those previously reported for cesium‐based halide perovskites, confirming the expected valence state of +1 for Cs in the lattice.^[^ ^28^ ^]^ A slight shift in the Cs peaks to higher binding energies was observed for the mixed‐halide analogs, Cs_3_Bi_2_Cl_4.5_Br_4.5_ and Cs_3_Bi_2_I_4.5_Br_4.5_, suggesting variations in the local electronic environment due to halide substitution effects,^[^ ^29^ ^]^ as reported in Figure 9S and 10S, Supporting Information. As expected, minimal variation of the binding energies was also observed in the case of the bromine atoms when conventional Cs_3_Bi_2_Br_9_ and L‐Cs_3_Bi_2_Br_9_ are compared. The Br 3*d*_5/2_ peak appears at ≈68.5 eV, in line with previously reported values for Bi–Br coordination.^[^ ^30^ ^]^ Notably, the binding energy differences between the 3*d*_3/2_ and 3*d*_5/2_ states of Cs and Br remained consistent with those of fully crystalline perovskite‐like materials, indicating the retention of a well‐defined electronic structure despite the different synthetic conditions.^[^ ^31^, ^32^ ^]^ A small shift to higher binding energy (≈0.5 eV) was detected in Cs_3_Bi_2_Cl_4.5_Br_4.5_, likely due to the increased electronegativity of Cl affecting the electronic distribution around Br atoms.^[^ ^33^ ^]^ The Cl 2*p*_3/2_ signal at ≈198.3 eV further confirms the successful incorporation of chloride within the perovskite lattice (Figure 9S, Supporting Information).^[^ ^34^ ^]^ The I 3*d*_5/2_ peak in Cs_3_Bi_2_I_4.5_Br_4.5_ was detected at 619.4 eV, comparable to iodine‐containing perovskites, further supporting the homogeneous integration of iodide within the structure (Figure 10S, Supporting Information).^[^ ^35^ ^]^

Regarding the metal component of the conventional Cs_3_Bi_2_Br_9_ material, the Bi 4*f* core‐level spectrum exhibits peaks at 160.8 eV (4*f*_7/2_) and 166.2 eV (4*f*_5/2_), indicative of the Bi(III) oxidation state, in agreement with literature values for bismuth halide perovskites (**Figure** **2**A).^[^ ^18^ ^]^ The variant shows the same peaks at lower binding energies (Figure 2B) and specifically at 159.3 eV (4*f*_7/2_) and 164.7 eV (4*f*_5/2_), suggesting a higher average electronic density for the surface metals in L‐Cs_3_Bi_2_Br_9_ material. This electronic characteristic is probably due to the surface vacancies of electronegative bromine atoms increasing the electron density of the surface bismuth atoms. The consistency of the Bi 4*f* XPS peaks across the mixed‐halide series confirms that halide exchange has a negligible impact on the bismuth oxidation state. Nonetheless, the emergence of a shoulder in Cs_3_Bi_2_I_4.5_Br_4.5_ (Figure 10S, Supporting Information) could be indicative of a non‐negligible contribution from elemental bismuth (Bi^0^),^[^ ^24^ ^]^ not evident in the Cs_3_Bi_2_Cl_4.5_Br_4.5_ counterpart (Figure 9S, Supporting Information).^[^ ^36^, ^37^ ^]^

The introduction of THAB during synthesis suppressed uncontrolled particle growth, resulting in smaller crystallites with an increased density of exposed catalytic sites, which is advantageous for enhancing photocatalytic performance. As demonstrated by diffuse reflectance spectra (Figure 2C), both Cs_3_Bi_2_Br_9_ materials exhibit similar absorption features with a sharp absorption profile in the case of the LARP‐modified PC.^[^ ^38^ ^]^ The Kubelka‐Munk transformation of the absorption spectra (Figure 2D) yields bandgap values of 2.60 eV for conventionally prepared Cs_3_Bi_2_Br_9_ and 2.57 eV for L‐Cs_3_Bi_2_Br_9_, indicating a slight decrease in the bandgap for the LARP prepared sample. This slight variation is likely due to subtle differences in particle size and surface states.^[^ ^21^, ^39^ ^]^ The analysis of Cs_3_Bi_2_Br_9_, Cs_3_Bi_2_Cl_4.5_Br_4.5_, and Cs_3_Bi_2_I_4.5_Br_4.5_ samples reveal that partial halogen substitution significantly influences their optical and electronic properties. **Figure** **3**A shows the absorption spectra of these perovskites: Substituting bromine with chlorine in Cs_3_Bi_2_Cl_4.5_Br_4.5_ results in an absorption onset around 446 nm, while substituting with iodine in Cs_3_Bi_2_I_4.5_Br_4.5_ shifts the onset to ≈582 nm. These shifts are attributed to the differing ionic sizes and electronegativities of the halogens, which affect the perovskite's electronic structure.^[^ ^40^ ^]^ Kubelka–Munk transformations of the absorption spectra, presented in Figure 3B, yield bandgaps of 2.60 eV for Cs_3_Bi_2_Br_9_, 2.78 eV for Cs_3_Bi_2_Cl_4.5_Br_4.5_, and 2.13 eV for Cs_3_Bi_2_I_4.5_Br_4.5_. These findings align with previous studies on halogen‐substituted perovskites, demonstrating that halogen substitution can effectively tune the electronic properties of these materials.^[^ ^41^ ^]^

XPS measurements (Figure 3C) quantify valence band maxima (VBM) at 2.51 eV for Cs_3_Bi_2_Br_9_, 2.92 eV for Cs_3_Bi_2_Cl_4.5_Br_4.5_, and 1.76 eV for Cs_3_Bi_2_I_4.5_Br_4.5_, respectively, in comparison to the normal hydrogen electrode (NHE) level. These shifts indicate that the chlorine introduction enhanced the oxidation potential of the relevant material (and potentially its photooxidation capabilities)^[^ ^42^ ^]^ due to the electronegativity of this atom. Conversely, iodine substitution lowers the VBM. The energy level diagrams in Figure 3D further highlight how halogen substitution influences the photocatalytic potential of these materials. As shown, Cs_3_Bi_2_Br_9_ exhibits an ideal bandgap of 2.60 eV, with VBM at 2.51 eV, and conduction band minima (CBM) at −0.09 eV. This electronic configuration enables efficient visible‐light absorption, with the CBM positioned favorably for photocatalytic processes. The VBM of Cs_3_Bi_2_Br_9_ aligns well with the oxidation potential of the BA/BAD couple (+1.98 eV vs. NHE),^[^ ^43^ ^]^ while the CBM matches the reduction potential for oxygen/superoxide couple (−0.33 eV vs. NHE),^[^ ^44^ ^]^ enabling its dual functionality in photooxidation and photoreduction processes.

The partial substitution of bromine with chlorine in Cs_3_Bi_2_Cl_4.5_Br_4.5_ increases the energy values of VBM to +2.92 eV and CBM to +0.14 eV, thus potentially enhancing the oxidation capabilities of the corresponding materials. Conversely, iodine substitution in Cs_3_Bi_2_I_4.5_Br_4.5_ lowers the VBM to +1.76 eV and the CBM to −0.37 eV. These results align with prior studies on halogen‐substituted perovskites, where halogen identity plays a significant role in modulating photocatalytic activity.^[^ ^45^, ^46^ ^]^

Cs_3_Bi_2_Br_9_, L‐Cs_3_Bi_2_Br_9_, and the two corresponding mixed‐halide analogs were analyzed by X‐ray powder diffraction (XRPD) data. For all of them, the full pathway of the ab initio structure solution process (i.e., indexing, space group determination, extraction of the integrated intensities, structure solution, and Rietveld refinement) was successfully carried out by *EXPO*.^[^ ^47^ ^]^ The four samples crystallized in the trigonal system (centrosymmetric space group *P*‐3*m*1); the table resuming the main crystallographic data for the four compounds is given in the Supporting Information (see Table 1S, Supporting Information). The structure models of Cs_3_Bi_2_Br_9_ and L‐Cs_3_Bi_2_Br_9_ were strongly similar, consequently, for the sake of brevity, additional details on the ab initio structure solution process will be summarized only for Cs_3_Bi_2_Br_9_ and its two corresponding mixed‐halides analogs; furthermore, only for these three samples, tables on the refined fractional atomic coordinates and isotropic displacement parameters, bond distances, and angles are provided in the SI (see Table 2S, 3S, and 4S, Supporting Information, respectively). The asymmetric unit of Cs_3_Bi_2_Br_9_ consists of five non‐H atoms (see Figure 11SA, Supporting Information, color setting by atomic species) and the crystal architecture shows layered arrangement of corner‐sharing BiBr_6_ polyhedra (see Figure 11SB and 12SB, Supporting Information). The final refined crystal structure of Cs_3_Bi_2_Br_9_ determined by *EXPO* was very similar to literature (see for example, the results obtained by Samanta et al.^[^ ^48^ ^]^ at ambient conditions, stored in the PDF‐5+ entry #01‐090‐9184):^[^ ^49^ ^]^ the root mean square deviation (RMSD) between the two structure models was 0.013 Å, where RMSD = sqrt(∑_i_ *d*_i_^2^/*N*_au_), i.e., the square root of the averaged squared distances between couples of corresponding atoms in the two compared models, with *N*_au,_ the number of atoms in the asymmetric unit. The final Rietveld refinement outcomes for Cs_3_Bi_2_Br_9_ are visualized in Figure 12SA, Supporting Information, showing the excellent match between observed (blue) and calculated (green) diffraction patterns, confirmed by the low value of the agreement factors *R*_p_ and *R*_wp_, i.e., 1.6% and 2.5%, respectively. In the case of the L‐Cs_3_Bi_2_Br_9_ material, the final refined crystal structure was very similar to that one obtained by applying the first protocol (the RMSD of the two crystal models was equal to 0.008 Å); the only relevant difference was the presence of a few low‐intensity‐impurity peaks in the powder diffraction pattern, probably responsible for a slight increase of the *R*‐factors at the end of the Rietveld refinement (i.e.*, R*_p_ and *R*_wp_ equal to 1.8% and 2.9% instead of 1.6% and 2.5%, respectively, see Table 1S, Supporting Information). This result confirms that the halide deficiency (experimentally measured by EDX) is mainly located at the surface of the L‐Cs_3_Bi_2_Br_9_ material not perturbing its bulk crystalline structure.

Also in the case of Cs_3_Bi_2_Cl_4.5_Br_4.5_ and Cs_3_Bi_2_I_4.5_Br_4.5_, the ab initio structure solution process by *EXPO* was successful; at the end of the Rietveld refinement, the agreement *R* factors *R*_p_ and *R*_wp_ were 1.8% and 2.7% for Cs_3_Bi_2_Cl_4.5_Br_4.5_ and 1.9% and 2.9% for Cs_3_Bi_2_I_4.5_Br_4.5_ and the final chemical formula was Cs_3_Bi_2_Cl_4.52_Br_4.48_ and Cs_3_Bi_2_I_4.56_Br_4.44_, respectively (see **Table** **1**), i.e., close to the expected formula corresponding to the equimolar ratio between the halides. The refined unit cell axes lengths for Cs_3_Bi_2_Br_9_, Cs_3_Bi_2_Cl_4.52_Br_4.48_ and Cs_3_Bi_2_I_4.56_Br_4.44_, are provided in Table 1, showing how *a* and *c* values change with the chemical composition; in particular, compared to the unit cell parameters of Cs_3_Bi_2_Br_9_, the *a* and *c* values for the corresponding mixed‐halides analogues decrease or increase, in case of presence of halogens lighter (i.e., Cl) or heavier (i.e., I) than Br, respectively, as expected, due to the different size of the halogens. In the case of Cs_3_Bi_2_I_4.56_Br_4.44_, the unit cell parameters are in agreement with the outcomes of Hodginks et al.,^[^ ^50^ ^]^ that explored a family of lead‐free perovskites Cs_3_Bi_2_Br_9‐*x*_I_x_, with 0 ≤ *x* ≤ 6, and observed: 1) an increase of the *a* and *c* parameters with increasing I content (i.e., with increasing *x* values); 2) the robustness of the layered topology of Cs_3_Bi_2_Br_9_ versus the iodide substitution (i.e., increasing the *x* values, the topology was the same up to a maximum value of *x* = 6); 3) whatever the investigated *x* value, the preference of I atoms to substitute one of the two sites shared with Br. The authors motivated the different site preference of the two halogens I and Br, by observing that I ions prefer the top and the bottom of the layered slabs, while Br ions preferentially occupy the bridging halogen positions within the inorganic layers, to reduce the strain within the lattice. Even if Hodginks et al. did not consider the present case (i.e., *x* ≈ 4.5), the refined lattice parameters obtained for Cs_3_Bi_2_I_4.56_Br_4.44_ follow a similar trend,^[^ ^50^ ^]^ being the unit cell axes lengths of Cs_3_Bi_2_I_4.56_Br_4.44_ larger than those ones obtained by Hodginks et al.^[^ ^50^ ^]^ in the case *x* ≈ 3.6 (see the entry # 04‐026‐2789 of the PDF‐5+ database in Table 1, with formula Cs_3_Bi_2_Br_5.35_I_3.65_) and smaller than those ones corresponding to the case *x* = 6 (see the entry # 04‐026‐2791 of the PDF‐5+ database^[^ ^49^ ^]^ in Table 1, with refined formula Cs_3_Bi_2_Br_3_I_6_). In addition, similarly to the outcomes of Hodginks et al.^[^ ^50^ ^]^ e.g., the refined crystal structures for Cs_3_Bi_2_Cl_4.5_Br_4.5_ and Cs_3_Bi_2_I_4.5_Br_4.5_ preserved the same layered topology of Cs_3_Bi_2_Br_9_ (by comparing their models with that one of Cs_3_Bi_2_Br_9_, the corresponding RMSD was 0.220 and 0.351 Å, for Cs_3_Bi_2_Cl_4.5_Br_4.5_ and Cs_3_Bi_2_I_4.5_Br_4.5_, respectively). Furthermore, as already found by Hodginks et al.^[^ ^50^ ^]^ for the I atoms of the lead‐free perovskites Cs_3_Bi_2_Br_9‐*x*_I_x_, in the case of Cs_3_Bi_2_Cl_4.5_Br_4.5_ and Cs_3_Bi_2_I_4.5_Br_4.5_, the heaviest atoms in the couples of halogens (I, Br) and (Cl, Br) preferred the top and the bottom positions in the slabs; this site preference was less pronounced for Cs_3_Bi_2_I_4.5_Br_4.5_, being the refined site occupancy factors (*SOF*) for the couple (I1, Br1) equal to 0.52(2) and 0.48(2), respectively (see Table 4S, Supporting Information); in the case of Cs_3_Bi_2_Cl_4.5_Br_4.5_, the refined *SOF* for (Br1, Cl1) were 0.640(7) and 0.360(7), respectively (see Table 3S, Supporting Information).

**Table 1:** Cs_3_Bi_2_Br_9_ and its corresponding mixed‐halides analogs (Cs_3_Bi_2_Cl_4.5_Br_4.5_ and Cs_3_Bi_2_I_4.5_Br_4.5_): unit cell axis lengths (*a*, *c*), cell volume (*V*), and final chemical formula (*FCF*, only in the case of the two mixed‐halides analogs of Cs_3_Bi_2_Br_9_) provided by *EXPO* at the end of the Rietveld refinement. In the last two columns on the right, the lattice parameters for two published crystal structures^[^ ^50^ ^]^ (together with the corresponding PDF‐5 + entries # in the table header) are given.

	Cs_3_Bi_2_Cl_4.5_Br_4.5_	Cs_3_Bi_2_Br_9_	Cs_3_Bi_2_I_4.5_Br_4.5_	PDF5‐entry # 04‐026‐2789	PDF5‐entry # 04‐026‐2791
*a*, *c* [Å]	7.7970(3), 9.5901(7)	7.95577(17), 9.8414(4)	8.2405(15), 10.113(4)	8.1626(1), 10.0316(1)	8.2881(1), 10.1679(2)
*V* [Å^3^]	504.90(4)	539.45(3)	594.7(3)	578.84(1)	604.88(2)
*FCF*	Cs_3_Bi_2_Cl_4.52_Br_4.48_	–	Cs_3_Bi_2_I_4.56_Br_4.44_	–	–

### 2.1 Photocatalytic Oxidation of Benzyl Alcohol

Photocatalytic tests on BA were conducted under visible‐light irradiation (>420 nm, 300 W Xe‐lamp) in air using the Cs_3_Bi_2_Br_9_ PC as the prototypical material for screening the best reaction conditions (**Table** **2**). The standard conditions were acetonitrile (ACN, 2.0 mL) and a PC load of 5.0 mg (3.3 mol% with respect to the substrate) for 3 h under stirring. Under these conditions, the conventionally prepared Cs_3_Bi_2_Br_9_ material exhibited a good conversion (77.4 ± 4.4%) with a BAD yield of 37.5 ± 0.3% (based on gas‐chromatography results, Figure 13S, Supporting Information). Although the product evolution rate under these conditions was remarkable (2500 μmol g^−^^1^ h^−^^1^), the selectivity toward BAD was found to be quite low (48.6 ± 0.6%). A kinetic study of photocatalytic conversion (and product evolution) was performed to assess the activity and stability of the PC. As shown in Figure 14S, Supporting Information, the reaction profiles exhibited typical kinetic behavior, confirming that the catalyst remains active and stable throughout the reaction cycle, achieving complete substrate conversion under the applied conditions. The same kinetic considerations can be extended to the L‐Cs_3_Bi_2_Br_9_ variant (vide infra). To ascertain the origin of the nonoptimal selectivity of the transformation under these conditions, we carried out the reaction without the PC (entry 2 of Table 2), observing the negligible BA conversion. This experiment not only confirms the crucial role of the PC in the reaction but also rules out the intrinsic light‐induced degradation of the BA substrate as the cause of the scarce reaction selectivity. Conversely, performing the reaction with the PC in the dark (entry 3 of Table 2) led to an apparent conversion (25.9 ± 5.2%), without the formation of the BAD product, highlighting the necessity of light activation for efficient photocatalysis. The residual conversion in the dark can be attributed to substrate adsorption on the catalyst surface, lowering the BA concentration in the reaction medium even if without its transformation. Conducting the reaction under an inert N_2_ atmosphere instead of air still resulted in a notable catalytic activity, surpassing the selectivity (92.1 ± 1.1%) observed under standard conditions (entry 4 of Table 2). This result suggests the occurrence of a relatively slower dehydrogenative mechanism (conversion of 54.9 ± 4.3% was observed after 3 h of reaction) under anaerobic conditions and probably concurrent with the conventional oxidative pathway under aerobic conditions. While molecular oxygen typically acts as the terminal electron acceptor in aerobic pathways, facilitating charge separation and sustaining the oxidation process, these outcomes indicate that, in its absence, BA oxidation can still take place through hydrogen evolution formed upon proton/electron coupling.

**Table 2:** Screening and control experiments of the photocatalytic oxidation of BA using the Cs_3_Bi_2_X_9_ materials synthesized in this study.

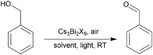				
Entry	Variation from the “standard conditions”a)	Conv. [%]b)	Yield [%]b)	Sel. [%]b)
1	None	77.4 ± 4.4	37.5 ± 0.3	48.6 ± 0.6
2	Without Cs_3_Bi_2_Br_9_	5.5 ± 5.8	1.1 ± 0.01	20.0 ± 0.2
3	In the dark	25.9 ± 5.2	–	–
4	N_2_ instead of air	54.9 ± 4.3	50.6 ± 0.6	92.1 ± 1.1
5	DCE instead of ACN	25.3 ± 5.2	3.0 ± 0.04	11.7 ± 0.1
6	DCM instead of ACN	15.2 ± 5.5	2.6 ± 0.03	16.8 ± 0.2
7	THF instead of ACN	17.0 ± 5.4	3.3 ± 0.04	19.5 ± 0.2
8	EtOAc instead of ACN	32.1 ± 5.0	7.5 ± 0.06	32.3 ± 0.4
9	CH instead of ACN	23.0 ± 5.3	8.4 ± 0.1	36.4 ± 0.4
10	CsPbBr_3_ instead of Cs_3_Bi_2_Br_9_	19.0 ± 5.4	–	–
11	Cs_3_Bi_2_I_4.5_ Br_4.5_ instead of Cs_3_Bi_2_Br_9_	30.6 ± 5.0	14.5 ± 0.2	47.3 ± 0.3
12	Cs_3_Bi_2_Cl_4.5_ Br_4.5_ instead of Cs_3_Bi_2_Br_9_	19.3 ± 5.4	–	–
13	L‐Cs_3_Bi_2_Br_9_	100.0	49.6 ± 0.4	49.6 ± 0.4
14	L‐Cs_3_Bi_2_Br_9_ (1.3%, 2 h)	75.8 ± 3.7	58.1 ± 0.7	76.6 ± 0.9
15	L‐Cs_3_Bi_2_Br_9_ (1.3%)	98.2 ± 3.0	78.1 ± 0.9	79.5 ± 0.9
16	L‐Cs_3_Bi_2_Br_9_ (1.3%, N_2_)	34.2 ± 4.9	1.0 ± 0.01	2.9 ± 0.04

a) Standard conditions: BA (0.1 mmol), Cs_3_Bi_2_Br_9_ (5 mg, 3.3 mol%), and ACN (2.0 mL) in air at room temperature, 300 W Xe‐lamp (>420 nm, 300 W) for 3 h.

b) GC yield%, conversion of BA and selectivity to BAD were determined by GC analysis using biphenyl as the internal standard.

Therefore, it can be anticipated that the light‐induced transformation mechanistically requires a substrate/PC interaction and that the process is occurring at the PC surface, while the relatively low selectivity in aerobic conditions can be explained by mineralization of the activated substrate in the presence of dioxygen. Unfortunately, low‐intensity (blue, green, or warm white) light sources (intentionally adopted to improve the selectivity) are inactive in the reaction, suggesting that a high concentration of photoinduced electron/hole couples is required to promote the substrate transformation independently of the reaction pathway (not shown in Table 2).

The impact of solvent choice was systematically investigated, as detailed in Table 2 (entries 5–9). Apart from ACN, all other explored solvents tested (dichloroethane, dichloromethane, tetrahydrofuran, ethyl acetate, and cyclohexane) were ineffective in the desired transformation (yields ≤8.4%). While emphasizing the critical role of solvent polarity and coordination ability in determining photocatalytic efficiency, these findings validate that ACN, employed under standard reaction conditions, enabled the highest conversion and yield probably due to its superior aptitude of stabilizing charge carriers.^[^ ^51^ ^]^

The effect of the PC composition was explored by replacing Cs_3_Bi_2_Br_9_ with alternative perovskite or bismuth perovskite‐like materials. Notably, when CsPbBr_3_ microparticles (prepared by the same synthetic protocol) were used (entry 10 of Table 2), no BAD evolution was observed. This behavior seems to align with previous studies, which attribute to the low efficiency of CsPbBr_3_ materials in photocatalytic reactions, including BA oxidation, to rapid charge recombination and unfavorable morphological characteristics.^[^ ^52^, ^53^ ^]^ The mixed‐halide compounds Cs_3_Bi_2_I_4.5_Br_4.5_ (entry 11 of Table 2) and Cs_3_Bi_2_Br_4.5_Cl_4.5_ (entry 12 of Table 2) also exhibited reduced activities. The negative performance of Cs_3_Bi_2_Br_4.5_Cl_4.5_ can be rationalized by the reduced light‐harvesting properties of the material. On the other hand, Cs_3_Bi_2_I_4.5_Br_4.5_ demonstrated modest yields (14.5 ± 0.2) likely due to nonperfect alignment of the VB energy level with the oxidation potential of BA/BAD couple (Figure 3D). Notably, the selectivity of this reaction was very similar to that of entry 1 of Table 1, suggesting the parasite reaction pathways are independent of the PC composition.

To optimize the reaction parameters, the variant of the Cs_3_Bi_2_Br_9_ congener, specifically the L‐Cs_3_Bi_2_Br_9_ PC, was introduced in the same reaction conditions. This PC exhibited enhanced performance in terms of conversion and yield compared to the conventionally prepared counterpart (entry 13 of Table 2). The superior performance can be attributed to the increased surface area for the generation of active species, which is a result of smaller particle sizes, and a different surface chemistry deriving from the halide vacancies. The selectivity, however, remained relatively low, suggesting the persistence of the same degradation pathways under aerobic conditions (Figure 14S, Supporting Information).

To improve the selectivity of the transformation, we attempted with a lower PC concentration (1.3% mol mol^−1^, entry 14 of Table 2) aiming at mitigating both the light scattering of the PC dispersion and the O_2_/reactive oxygen species (ROS) distribution at its surface, where an excess ROS can tentatively be ascribed as responsible for the activation of the degradation pathways (Scheme 1S, Supporting Information). While no changes in the product evolution rate were observed for the conventionally prepared material upon modification of the PC concentration, a lower catalyst load for the L‐Cs_3_Bi_2_Br_9_ material resulted in 5.8‐fold higher production rate (14 525 μmol g^−^^1^ h^−^^1^). This impressive result^[^ ^54^ ^]^ is a direct consequence of the enhanced selectivity (76.6 ± 0.9%) which remained high also after 3.0 h of reaction (entry 15 of Table 2). Surprisingly, the reaction carried out under an inert atmosphere (entry 16 of Table 2) did not promote the BAD evolution confirming the different nature of the surface composition of the L‐Cs_3_Bi_2_Br_9_ catalyst. Probably, the anaerobic transformation with the halide‐deficient catalyst surface is hampered by the absence of bromine atoms near to the benzyl position for the hydride shift (vide infra).

### 2.2 Oxidation of Substituted Benzyl Alcohols

The scope of photocatalytic oxidation at the benzyl position was broadened to showcase the versatility and extensive applicability of L‐Cs_3_Bi_2_Br_9_ PC in air, as detailed in **Table** **3**. The findings reveal that electron‐donating groups (such as –OCH_3_ and –CH_3_) enhance the oxidation process, resulting in faster conversion rates (entries 1 and 2 of Table 3). In fact, 4‐methylbenzyl alcohol (entry 1 of Table 3) was completely converted to 4‐methylbenzaldehyde (67.0% isolated yield) within 3.0 h. However, the detection of isophthalaldehyde (qualitatively identified by gas‐chromatography (GC) analyses prior to purification) indicates the presence of competing radical degradation pathways, which adversely affect the yield of the target product. Similarly, 4‐methoxybenzyl alcohol (entry 2 of Table 3) underwent selective oxidation to 4‐methoxybenzaldehyde, achieving a remarkable yield of 88.0% within the same reaction time with no significant and detectable side products. This observation underscores the significance of electron‐donating groups in enhancing the oxidation reaction rate, likely due to the stabilization of the resulting benzyl radical intermediates (vide infra). Concurrently, the reaction rates for *p*‐substituted substrates with electron‐withdrawing groups (–F, –Cl, –Br, –NO_2_) were substantially enhanced, achieving complete conversion within 3.0–4.0 h (entries 3‐6 of Table 3). Likely, in this instance, the formed benzyl radical (an intermediate in the transformation, vide infra) was stabilized by *p*‐substituents, thereby accelerating the reaction rates. These reactions were consistently characterized by the formation of corresponding benzoic acids as by‐products. While this did not affect the yields (72.6%–87.0%), it indicates that electron‐withdrawing groups activate the aldehyde products through radical pathways, requiring the reaction to be halted to preserve high selectivity. Thus, we can conclude that, except for the methyl group, the other common functionalities remain intact under the proposed reaction conditions.

**Table 3:** Substrate scope using L‐Cs_3_Bi_2_Br_9_ as PC.

Entry	Substrate	Conversion time [h]	Expected product	Yield [%]a)	Side product [s]
1	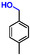	3.0	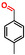	67.0	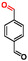
2	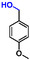	3.0	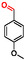	88.0	–
3	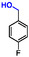	3.0	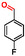	80.0	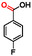
4	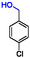	4.0	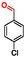	86.1	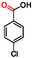
5	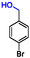	3.0	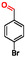	87.0	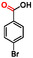
6	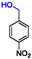	4.0	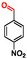	72.6	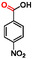
7	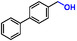	8.0	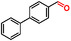	75.7	–
8	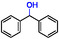	3.0	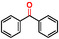	68.0	–
9	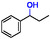	3.5	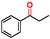	70.0	–
10	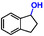	4.5	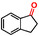	46.4	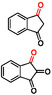

Reaction conditions: substrate (0.1 mmol), L‐Cs_3_Bi_2_Br_9_ (1.3% mol mol^−1^), and ACN (2.0 mL) in air at room temperature, 300 W Xe‐lamp (>420 nm, 300 W).

a) Isolated yield. Conversion and side products determined by GC‐MS analyses.

To assess the impact of steric hindrance, 4‐phenylbenzyl alcohol was subjected to the oxidation process, taking 8.0 h for complete conversion (entry 7 of Table 3). This duration is notably longer than that required for simpler BAs, indicating reduced benzyl radical formation due to weaker interactions between the substrate and the catalyst. Despite the prolonged reaction time, 4‐phenylbenzaldehyde was obtained in good yield (75.7%) and no side products were detected. Conversely, the isomer diphenylmethanol (entry 8 of Table 3) underwent oxidation to benzophenone with a good yield (68.0%) in 3.0 h, without any detectable side products. This suggests that the high stabilization of the benzyl radical (a secondary carbon with two phenyl groups) significantly accelerates the oxidation process, despite the increased steric encumbrance of the substrate. The selective oxidation of a secondary carbon was also conducted using 1‐phenyl‐1‐propanol as the substrate (entry 9 of Table 3), resulting in 1‐phenyl‐1‐propanone with a satisfactory yield of 70.0% within 3.5 h and thus emphasizing the role of steric effects on the reaction rates. Conversely, 1‐indanol (entry 10 of Table 3) underwent oxidation to 1‐indanone, with 1,3‐indandione and 1,2,3‐indantrione as side products, which drastically lowered the yield of the reaction to 46.4%, despite achieving total conversion in 4.5 h. Likely, the conformational constraints of the condensed structure of this substrate favor oxidation at the benzyl position deriving from a radical process, thereby affecting the overall selectivity of the process.

Finally, alcohols containing heteroaromatic scaffolds, such as 3‐pyridinemethanol and indolyl methanol, exhibited poor conversion (not shown in Table 3). This behavior is likely due to the coordination of the heteroatom (nitrogen) to the PC surface, which can hinder (not stabilizing the corresponding anion or radical) the hydrogen abstraction step crucial for the oxidation process (vide infra). Consequently, this interaction leads to reduced reaction efficiency and lower yields, emphasizing the importance of substrate/PC interactions in controlling the photocatalytic oxidation process.

### 2.3 Mechanistic Insights and Computational Studies

Oxidative reactions in the presence of ROS can also be accompanied by concurrent degradation pathways, induced by the delocalization of the benzyl radical, considered a plausible intermediate of the reaction. The oxygen attack to different positions of the substrate scaffold is likely responsible for the relatively low selectivity of the reaction under high concentration of ROS at the semiconductor surface.^[^ ^55^ ^]^ To indirectly demonstrate the role of mineralization as a competitive pathway in oxidation, we conducted the reaction using anthracene‐9‐methanol as the substrate. Under the same reaction conditions, the predominant formation of 9,10‐anthraquinone along with the expected aldehyde was recorded as a minor product. The remote oxidation to the 10‐position of the substrate suggests that benzyl substrates are prone to alternative degradation pathways, leading to overoxidation and/or mineralization under radical mechanisms. As evidenced in Scheme 1S, Supporting Information, the anthracene scaffold preserves the aromaticity of the adjacent systems hampering the overoxidation plausibly observed for simpler aromatic substrates. The competitive mineralization process not only reduces the BAD yield but also suggests that careful control over the oxidative species formed during the reaction is crucial to achieving high selectivity for the desired aldehyde.

To rule out the plausible mediation of singlet dioxygen in the oxidative transformation of benzyl scaffolds (or degradation of anthracene‐9‐methanol), the reactivity of furan‐based derivatives, such as furan‐2‐ylmethanol and furan‐2,5‐diyldimethanol, was explored. In fact, the peculiarity concerning the absence of emission in the investigated perovskite systems does not totally preclude the possibility of dioxygen sensibilization, while furan derivatives are known to undergo rapid reactions with singlet dioxygen, forming endoperoxides as major products of [4 + 2] cycloaddition reactions, which can lead to degradation pathways alternative to the desired oxidation process.^[^ ^56^ ^]^ However, the negligible conversion of these furan‐based alcohols suggests not only the absence of singlet dioxygen in the reaction medium but also that their structure plausibly interferes with the target transformation. It is reasonable to infer that the furan electron‐withdrawing oxygen atom entraps the unpaired electron of the forming radicals, thereby hindering the generation of the key intermediate (benzyl radical following the hydrogen abstraction) for the substrate conversion.^[^ ^57^, ^58^ ^]^ As in the case of nitrogen‐containing compounds, these findings further indicate that photocatalytic oxidation pathways is strongly influenced by the electronic properties of the substrate.^[^ ^59^ ^]^

In contrast, BA undergoes efficient oxidation via a radical‐based mechanism, facilitated by proton abstraction and benzyl radical formation (vide infra). To experimentally support the involvement of hole transfer in the reaction pathway, we investigated the effect of hole scavenger. The addition of ammonium formate resulted in a significant decrease in conversion (28.5 ± 5.1%) with no detectable formation of the target aldehyde. This observation strongly supports a radical‐driven oxidation mechanism, as ammonium formate is known to quench photogenerated holes, suppressing key oxidative steps required for benzyl radical formation.^[^ ^60^, ^61^, ^62^ ^]^ The lack of benzaldehyde formation in our system further corroborates that the reaction proceeds involving a hole‐mediated pathway.

To gain a deeper understanding of the catalytic pathway of BA oxidation promoted by (Bi_2_Br_9_)^3−^ or (Bi_2_Br_8_)^2−^—simulating the halide‐deficient surfaces—clusters, density functional theory (DFT) calculations were performed using LANL2DZ/6‐311 G+(d,p) basis sets and the M06‐2X functional including the conductor‐like polarizable continuum model (CPCM, ACN) for the solvation modeling of the Gaussian09 suite. Computational studies are intended to provide constructive insights into the reaction pathways by discriminating them as a function of the PC's surface atoms and the presence or not of ROS (i.e., aerobic and anaerobic conditions) in the reaction medium, as experimentally observed. Concerning the surface, the substrate was interacting with the [Bi_2_Br_9_]^3−^ cluster via a hydrogen bond^[^ ^63^, ^64^ ^]^ or with the [Bi_2_Br_8_]_2_^2−^ cluster via the Bi–O bond formation according with the relevant adsorption aptitude of the benzylic substrate for the used PCs of this study (vide supra). In the first case (aerobic oxidation with halide‐rich surfaces, **Figure** **4**A), the initial state (IS) was attacked by the superoxide anions (formed after electron injection to dioxygen) acting as a base removing the benzylic protons of the substrate (generating INT1). Even if the target of the superoxide anions can also be the proton of the –OH group, its abstraction results to be not inherent with the substrate evolution. Although thermodynamically unfavored (+51.93 kcal mol^−1^), this process can occur due to the high concentration of ROS at the PC surface but can be considered the rate determining step judging from the entire energy profile of the transformation. The oxidation pathway starts with photoinduced hole injection from the excited PC into the deprotonated BA, yielding the corresponding benzyl radical intermediate (INT2, +47.71 kcal mol^−1^). This reactive intermediate can readily couple with molecular oxygen adsorbed on the PC surface, leading to the formation of peroxidic species (INT3). The computed free energy change associated with this process (+33.96 kcal mol^−1^) indicates that the benzyl radical/O_2_ interaction is highly favorable, promoting the conversion of the radical intermediate into BAD (final state, FS). Its evolution involves a concerted 5‐members transition state with an activation energy (17.44 kcal mol^−1^) compatible with room‐temperature transformations.

The parallel dehydrogenative pathway, operative under anaerobic‐like conditions, was systematically investigated. In this route, BA is directly oxidized to BAD through interfacial hydrogen atoms transfer with the PC surface, effectively bypassing the formation of free radical intermediates. The initial state (IS‐cat) illustrated in Figure 4B is assigned to the catalyst–substrate complex generated upon photoinduced hole injection. The complete suppression of reactivity in the presence of ammonium formate, in fact, corroborates the assumption that this reaction pathway necessarily involves the hole injection into one of the substrate intermediates and not only the electron‐transfer to dioxygen.

The transition state (TS2) calculations reveal a slightly lower energy barrier (13.32 kcal mol^−1^), indicating that the hydride shift at the PC surface (through electronegative bromine atoms) is kinetically comparable to that of the dioxygen‐mediated mechanism. The hydrogen abstraction from the hydroxyl groups is conversely the rate determining step of the process with an activation energy of 27.30 kcal mol^−1^, leading to the BAD evolution (FS‐cat). This means that the anaerobic process is effectively slower (but more selective, due to the absence of an external oxidant) than the aerobic one. The two hydrogen atoms removed from the substrate are thought to plausibly capture photoinduced free electrons producing dihydrogen and consequently restoring the PC.

The energy profile of the BA transformation at the halide‐vacancy (or metal‐rich) surface is shown in Figure 4C. The IS of this pathway (IS‐hv) evolves into the corresponding anion (INT5, +41.75 kcal mol^−1^) through a thermodynamically unfavored benzyl hydrogen abstraction, which, however, is remarkably energetically more feasible than the INT1 formation. This means that Bi–O secondary bond (the probable way through which catalyst and substrate interact) stabilizes the corresponding anion leading to an improvement of the PC performances even in lower reaction loading. While the energy profile of oxidation (INT6) and consequent dioxygen addition (INT7) follows the same trend observed in Figure 4A, the effect of the different PC/substrate interactions is evident also in the case of the TS computation (TS4 in Figure 4C), which shows a relatively lower activation energy (16.04 kcal mol^−1^) hinting that the best performance of L‐Cs_3_Bi_2_Br_9_ PC is also due to the minor energy demand for the BAD evolution (FS‐hv) mediated by dioxygen.

The reaction pathway was also investigated in the absence of a catalytic surface, commencing from the computed energy of the BA species (IS‐free, Figure 4D). In analogy with the previously analyzed catalytic scenarios, the initial step (hydrogen abstraction from BA mediated by the superoxide anion) was found to be thermodynamically unfavorable (INT8 = +45.69 kcal mol^−1^). Notably, the radical intermediate formed via photooxidation at the PC interface (INT9) exhibits a significantly high energy level (+109.65 kcal mol^−1^), indicative of its pronounced thermodynamic instability in comparison to PC‐mediated pathways. While the dioxygen attack to the radical intermediate lowers the energy of the system of 14.32 kcal mol^−1^ (INT10), the calculated activation energy (TS5 = 11.92 kcal mol^−1^) for the conversion of BAD (FS‐free) suggests that the oxidation process proceeds with a reduced energy barrier in comparison to surface‐mediated interactions.

## 3 Conclusions

We demonstrate that Cs_3_Bi_2_Br_9_ is an efficient, low‐toxicity PC for the selective oxidation of BA under mild, visible‐light conditions. The success of Cs_3_Bi_2_Br_9_ is attributed to its optimal bandgap and favorable energy level alignment, which outperforms other metal halide perovskite materials. Synthesizing the material under halide‐rich conditions yields smaller Cs_3_Bi_2_Br_9_ crystallites exposing more metal centered catalytic sites (deriving from the removal of quaternary ammonium salt during washing cycles). These microparticles achieve a record product evolution rate of 14,525 μmol g^−^^1^ h^−^^1^ for heterogeneous BA oxidation. The photocatalytic method is versatile, accommodating various functional groups on the benzylic substrate and enabling ketone formation from secondary alcohols. Theoretical calculations reveal that the superoxide anion initiates photoinduced oxidation by extracting a hydrogen atom from the benzyl position, a more favorable step for halide‐deficient catalyst surfaces. These insights clarify how surface halide stoichiometry controls active‐site exposure and reaction selectivity. They provide a roadmap for engineering next‐generation, lead‐free perovskite‐based PCs for sustainable fine‐chemical synthesis.

## 4 Experimental Section

4.1

4.1.1

##### Synthesis of Cs_*3*_ *Bi*_*2*_ *X*_*9*_ (X = Cl, Br, I) Microcrystals

Conventional Cs_3_Bi_2_Br_9_ particles were synthesized via an antisolvent reprecipitation method. CsBr (0.45 mmol, 96.0 mg) and BiBr_3_ (0.30 mmol, 134.5 mg) were dissolved in DMSO (10 mL) under continuous stirring at room temperature. The resulting solution was rapidly injected into vigorously stirred isopropanol (200 mL), leading to immediate precipitation of a yellow solid. The product was collected by centrifugation (4000 rpm, 10 min), washed with ethanol (3 × 20 mL), and dried under vacuum at 50 °C for 24 h, yielding 183 mg of Cs_3_Bi_2_Br_9_. Mixed‐halide analogs, Cs_3_Bi_2_I_4.5_Br_4.5_ and Cs_3_Bi_2_Cl_4.5_Br_4.5_ were prepared using the same procedure, replacing CsBr and BiBr_3_ with equimolar amounts of CsI/BiI_3_ and CsCl/BiCl_3_, respectively. Final products were washed and dried as above, affording 150 mg and 175 mg of product, respectively.

##### Synthesis of L‐Cs_3_Bi_2_Br_9_ Microcrystals

CsBr (0.45 mmol, 96.0 mg), BiBr_3_ (0.30 mmol, 134.5 mg), and THAB (0.15 mmol, 65.2 mg) were dissolved in DMSO (10 mL) under stirring. The solution was injected into isopropanol (200 mL) under vigorous stirring, and the yellow precipitate was isolated by centrifugation, washed with ethanol (3 × 20 mL), and dried under vacuum at 50 °C to afford 180 mg of L‐Cs_3_Bi_2_Br_9_ as a yellow solid.

##### General Procedure for The Photocatalytic Oxidation of Benzyl Alcohols

Photocatalytic oxidation reactions were carried out in 5 mL borosilicate glass tubes (12 mm diameter, 75 mm length) containing L‐Cs_3_Bi_2_Br_9_ (1.3% mol mol^−1^ relative to substrate, 2 mg), BA (0.1 mmol, 10.4 μL), biphenyl as internal standard (0.1 mmol, 15.4 mg), and ACN (2 mL). The reaction mixture was stirred and irradiated using a 300 W xenon arc lamp (LS300Xe, Quantum Design Europe) placed at a fixed distance of 10 cm from the sample. A UV long‐pass filter (λ > 420 nm) was employed to ensure visible‐light‐only activation. Reactions with conventionally prepared Cs_3_Bi_2_Br_9_ were performed under the same conditions using 5 mg of catalyst (corresponding to 3.3% mol mol^−1^). Product yields and conversions were determined by GC‐MS analyses using biphenyl as internal standard (Figure 12S, Supporting Information). In the case of isolated yields, after complete conversion determined by GC analyses, the catalyst was removed by centrifugation. The supernatant was concentrated under reduced pressure, and the crude mixture was purified by flash column chromatography using the appropriate eluent (*n*‐hexane/ethyl acetate mixtures).

## Conflict of Interest

The authors declare no conflict of interest.

## Supporting information

Supplementary Material

## Data Availability

The data that support the findings of this study are openly available in [Cambridge Crystallographic Data Centre] at [https://www.ccdc.cam.ac.uk/structures], reference number [2473376].
