# Supplementary material for: Lighting the Way: Unveiling the Mechanisms of the Photoinduced Benzyl Alcohol Oxidation Using Tailored Bismuth‐Based Perovskite‐Inspired Microcrystals
Source: ChemSusChem. 2025 Sep 18;18(20):e202501555. doi: 10.1002/cssc.202501555 (PMC12548941; doi:10.1002/cssc.202501555)
Supplement: Supplementary file 1 — Supplementary Material [file CSSC-18-e202501555-s001.pdf]

SUPPORTING INFORMATION

Lighting the way: unveiling the mechanisms of the photoinduced benzylic alcohol oxidation via tailored bismuth-based perovskite microcrystals

Daniele Conelli,[a] Chiara Lo Porto,[a] Anna Moliterni,[b] Davide Altamura,[b] Cinzia Giannini,[b] Fabio Palumbo,[c] Helena Mateos,[d] Mokurala Krishnaiah,[e] Tuhin Samanta,[e] Kimmo Lahtonen,[f] G. Krishnamurthy Grandhi,[e] Paola Vivo,[e] Gian Paolo Suranna[a,g] and Roberto Grisorio*[a]*

[a]Dipartimento di Ingegneria Civile, Ambientale, del Territorio, Edile e di Chimica (DICATECh), Politecnico di Bari, Via Orabona 4, 70125 Bari, Italy. E-mail: roberto.grisorio@poliba.it

[b]CNR–Istituto di Cristallografia, via G. Amendola 122/O, 70126 Bari, Italy.

[c]National Research Council, Institute of Nanotechnology (CNR-NANOTEC), c/o Department of Chemistry, University of Bari ''Aldo Moro'', via Orabona 4, Bari 70125, Italy.

[d]Dipartimento di Chimica and CSGI (Center for Colloid and Surface Science), Università degli Studi di Bari ''Aldo Moro'', via Orabona 4, 70125 Bari, Italy.

[e]Hybrid Solar Cells, Faculty of Engineering and Natural Sciences, P.O. Box 541, FI-33014 Tampere University, Finland.

[f]Faculty of Engineering and Natural Sciences, P.O. Box 541, FI-33014 Tampere University, Finland.

[g]CNR-NANOTEC – Institute of Nanotechnology, c/o Campus Ecoteckne, Via Monteroni, 73100 Lecce, Italy.

Chemicals: Cesium bromide (CsBr, 99.999% trace metals basis, Aldrich), cesium chloride (CsCl, 99.999% trace metals basis, Aldrich), cesium iodide (CsI, 99.999% trace metals basis, Aldrich), lead bromide ($PbBr_2$, 99.999% metals basis, Aldrich), lead chloride ($PbCl_2$, 99.999% metals basis, Aldrich), lead iodide (PbI_2, 99.999% metals basis, Aldrich), bismuth(III) bromide ($BiBr_3$, ≥98%, Aldrich), bismuth(III) chloride ($BiCl_3$, ≥98%, Aldrich), bismuth(III) iodide (BiI_3, ≥98%, Aldrich), dimethyl sulfoxide (DMSO anhydrous, ≥99.9%, Aldrich), tetrahexylammonium bromide (THAB, 99%, Aldrich), 2-propanol (IPA, Honeywell Riedel-de Haën, ≥99.8% GC grade), acetonitrile (ACN, ≥99.9%, Aldrich, GC grade), ethyl acetate (EA, VWR Chemicals BDH, ≥99.5%, ACS), ethanol absolute (VWR Chemicals BDH, ≥99.8%, ACS), 1,2-dichloroethane (DCE, ≥99.5%, EMPLURA, GC grade), dichloromethane (DCM, ≥99%, EMPLURA, GC grade), tetrahydrofuran (THF, ≥99.9%, anhydrous, Aldrich), cyclohexane (CH, ≥99%, EMPLURA, GC grade), dimethyl carbonate (DMC, ReagentPlus, 99%), toluene (TOL, ACS reagent, ≥99.5%), benzyl alcohol (anhydrous, 99.8%). p-methylbenzyl alcohol (≥98%, Aldrich), p-methoxybenzyl alcohol (≥98%, Aldrich), p-fluorobenzyl alcohol (≥98%, Aldrich), p-chlorobenzyl alcohol (≥98%, Aldrich), p-bromobenzyl alcohol (≥98%, Aldrich), p-nitrobenzyl alcohol (≥98%, Aldrich), p-phenylbenzyl alcohol (≥98%, Aldrich), 1-indanol (≥98%, Aldrich), 9-anthracenemethanol (≥98%, Aldrich), 1-phenyl-1-propanol (≥98%, Aldrich), diphenylmethanol (≥98%, Aldrich), 3-pyridinemethanol (≥98%, Aldrich), (1H-indol-2-yl)methanol (≥98%, Aldrich), furan-2-ylmethanol (≥98%, Aldrich), and furan-2,5-diyldimethanol (≥98%, Aldrich). All chemicals were used as received without further purification.

Instrumental facilities: The morphology of the powders was acquired by scanning electron microscopy coupled with energy dispersive X-ray spectroscopy using a Zeiss Σigma 300VP electron microscope equipped with an Oxford C-MaxN SDD detector with an active area of 20 mm^2. Perovskite samples were deposited on aluminum stubs and the images were recorded at

working distance of 7.5 mm, an acceleration voltage of 15 kV and a magnification of 1000×. The analysis accuracy was checked using the MAC (Micro-Analysis Consultants Ltd) reference materials.

The thermogravimetry was carried out using a Q600 TA instrument under a nitrogen flow (40 mL/min) at a temperature scan of 10 °C/min.

A first set of X-ray Photoelectron Spectroscopy (XPS) measurements was carried out with a PHI 5000 Versa Probe II spectrometer (Physical Electronics) equipped with a monochromatic Al Kα X-ray source (1486.6 eV), operated at 15 kV and 24.8 W, with a spot size of 200 µm. Survey (0–1200 eV) and high-resolution spectra were recorded in Fixed Analyzer Transmission mode at a pass energy of 117.40 eV, 29.35 eV, respectively. Surface charging was compensated by means of a dual beam charge neutralization system, with a combination of a flux of low energy electrons (∼1 eV) and a one of low energy Ar^+ ions (10 eV). The correction for the binding energies charging was carried out with respect to the C 1s core level line at 285.0 eV. All spectra were collected at an angle of 45° with respect to the sample surface. Curve fitting of C 1s was carried out with MultiPak™ data processing software (Physical Electronics), using a Shirley background, a 80% Gaussian/Lorentzian peak shape and a FWHM of 1.4 eV.

For a second set of measurements, an excitation source utilizing non-monochromatic Al Kα radiation (hν = 1486.6 eV), generated by the ESCA3000 CLAM4 electron spectrometer (VG Microtech)was used for XPS measurements. Data acquisition is done with VGX900 version 8.4-A-A (Scientific Instrument Consultants. The processing and analysis were performed using CasaXPS version 2.3.22 PR1.0. Peak fitting was carried out after Shirley background subtraction, employing a Gaussian–Lorentzian function to approximate the line shapes of the fitted components. Calibration of the spectra was achieved by setting the C 1s (C–C/H) peak to 284.8 eV. The surface composition of the samples was determined by analyzing core-level photoelectron transitions using X-ray Photoelectron Spectroscopy (XPS). Selected-area XPS measurements were conducted over a

~0.6 mm region using an XR3E2 Al Kα X-ray source and an ESCA3000 CLAM4 electron spectrometer (VG Microtech). The acquired spectra were corrected for the spectrometer transmission function (TRF) and had the Shirley background subtracted. Spectral fitting was performed using CasaXPS software (version 2.3.25PR1.0), applying Gaussian–Lorentzian (GL) line shapes.[S1] The minimum number of synthetic components necessary for a good fit was used. Binding energy calibration was referenced to the C 1s (C–C/H) peak at 284.8 eV. Photoelectron transition-specific relative sensitivity factors (RSFs) were derived from Scofield photoionization cross sections.[S2] Relativistic inelastic mean free paths (IMFPs) of electrons were calculated using the TPP-2M formula. Semi-quantitative atomic concentrations of elements in various chemical states were calculated using the formula:[S3]

$$\text{Concentration} \propto A / (\text{TRF} \times \text{RSF} \times \text{IMFP})$$

where A is the raw peak area of each fitted component.

Note that the binding energy (BE) scale calibration was performed differently for the Physical Electronics and VG Microtech XPS data, making the spectral positions not directly comparable. This calibration difference introduces a shift of approximately 0.2 eV, which should be corrected—or at least acknowledged—when comparing spectra obtained from different instruments.

The diffuse reflectance spectroscopy measurements of the powder samples were acquired on a Jasco V670 spectrophotometer, which is equipped with an integrating sphere.

The Dynamic Light Scattering (DLS) and zeta potential measurements were acquired using Malvern Zetasizer Advance ULTRA instrument using ZS XPLORER 3.3.1.5 Software. Powder samples were dispersed in 94% ethanol with the help of a Vortex instrument. We used the following parameters to define the solvent at measurement temperature (25 °C): Dielectric constant 24.3, Viscosity: 1.040 mPa·s, Refractive index 1.361. The DLS measurements are particularly tricky in the case of micron size particles. This is because very large particles have a slow Brownian motion

but a large cross section that makes them strongly influenced by these convective currents. To overcome this problem the sizing measurements have been performed in low volume capillaries (ZSU003 Malvern) that ensure negligible convection allowing to extend the upper particle size range to 10 μm measuring the scattering at 90° angle. Laser Doppler Electrophoresis (LDE) measurements have been performed with the same instrument in disposable folded capillary cells (Malvern #DTS1070). The zeta-potential was subsequently evaluated from the electrophoretic mobility measured by LDE according to the Hückel approximation

XRPD data were collected on a Rigaku RINT2500 rotating anode diffractometer (50 kV, 200 mA) equipped with a silicon strip Rigaku D/teX Ultra detector. An asymmetric Johansson Ge(111) crystal was used to select the monochromatic Cu Kα1 radiation (λ= 1.54056 Å). Measurements were carried out in transmission mode by introducing the powder in a Lindemann glass capillary tube with a diameter of 0.5 mm. The XRPD patterns were recorded in the 2θ range 5-120° (for $Cs_3Bi_2Br_9$) and 10−120° (for its mixed-halides analogues $Cs_3Bi_2Cl_{4.5}Br_{4.5}$ and $Cs_3Bi_2I_{4.5}Br_{4.5}$) by step scanning, using 2θ increments of 0.02° and a fixed counting time of 2 sec/step.

TEM images were acquired using JEM-F200 (200 kV) drop-casting a diluted dispersion of the sample on a carbon-coated Cu grid. The solvent was left to evaporate for several minutes, after which the grid was loaded into the TEM and imaged.

The substrate conversion and the product evolution were determined by GC–MS (EI, 70 eV) performed on an HP 6890 instrument equipped with an HP-5MS 5% phenyl methyl siloxane (30.0 m × 250 μm × 0.25 μm) coupled with an HP 5973 mass spectrometer passing from 50 °C to 280 °C with a ramp of 15 °C/min.

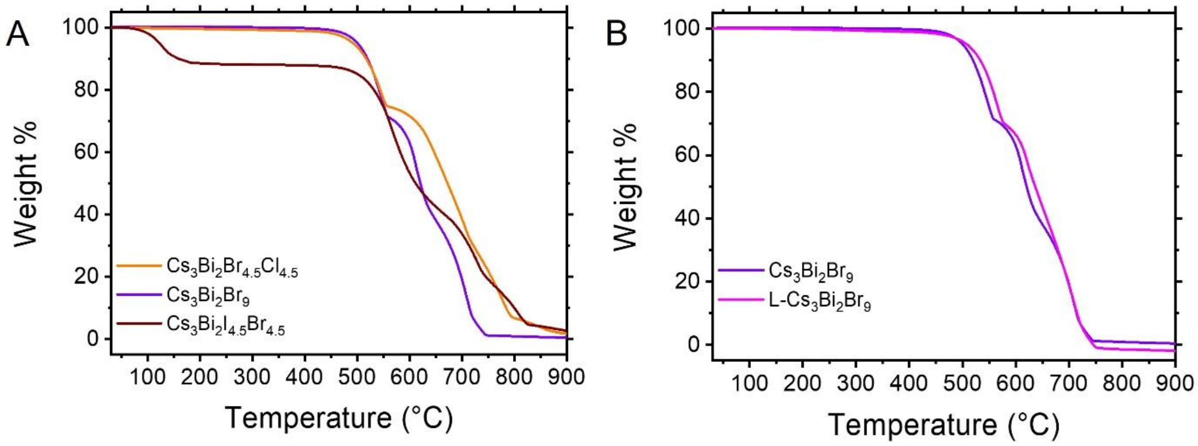

Figure 1S. TGA profiles of the materials (A) synthesized with the conventional approach (B) and the comparison between bulk $Cs_3Bi_2Br_9$ and L-$Cs_3Bi_2Br_9$ materials.

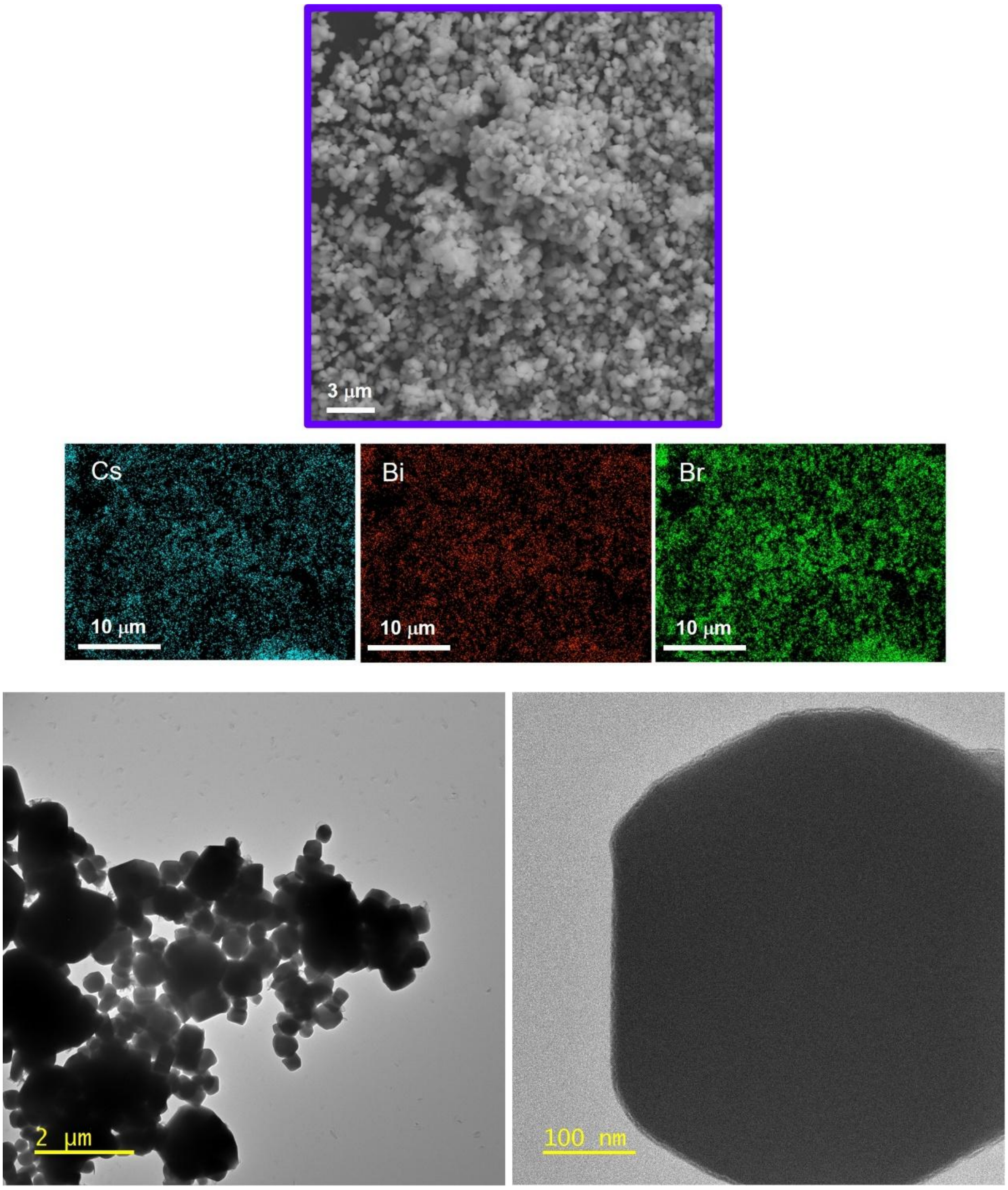

Figure 2S. SEM image, EDX mapping of the elements present in the $Cs_3Bi_2Br_9$ material and additional TEM images.

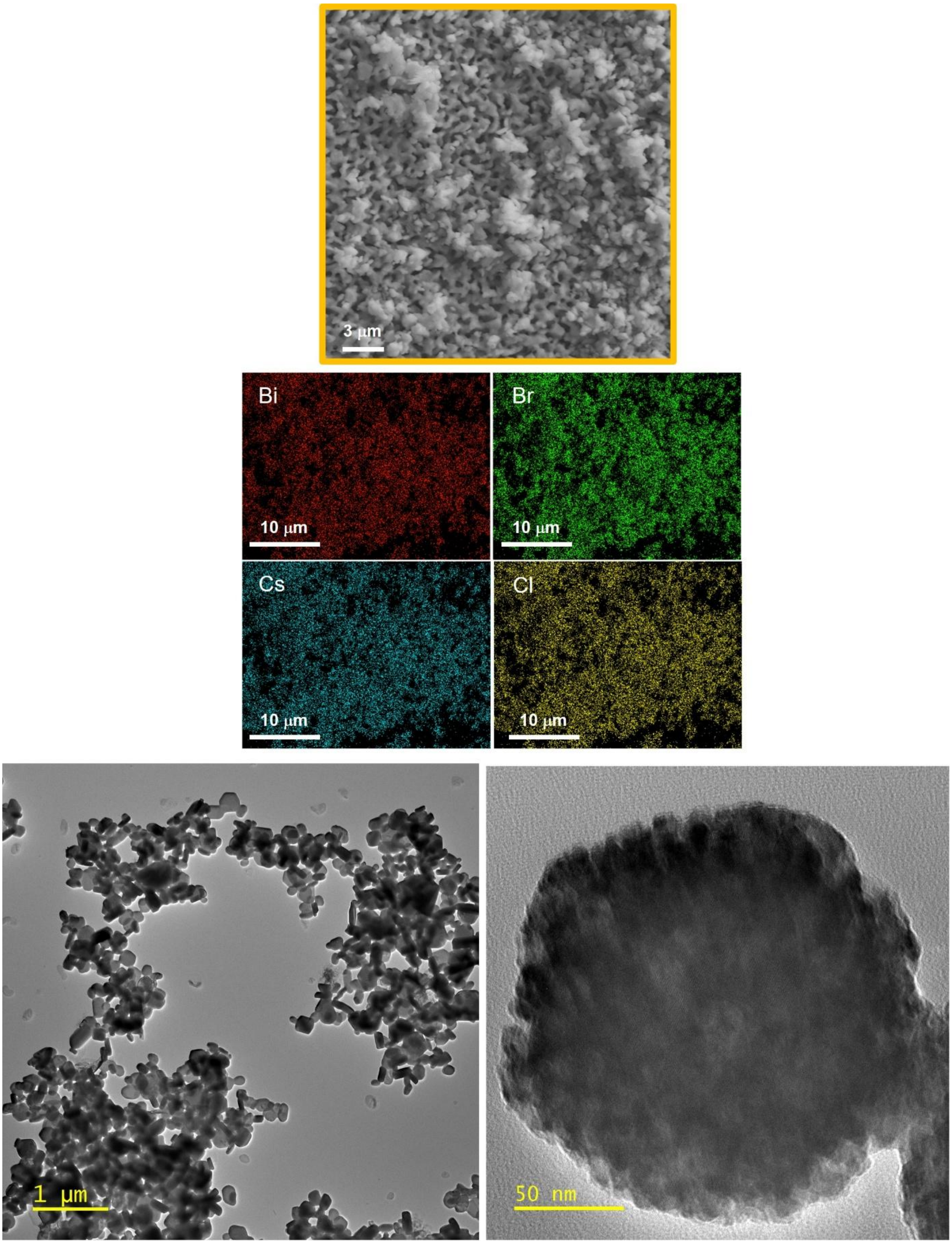

Figure 3S. SEM image, EDX mapping of the elements present in the $Cs_3Bi_2Br_{4.5}Cl_{4.5}$ material and additional TEM images.

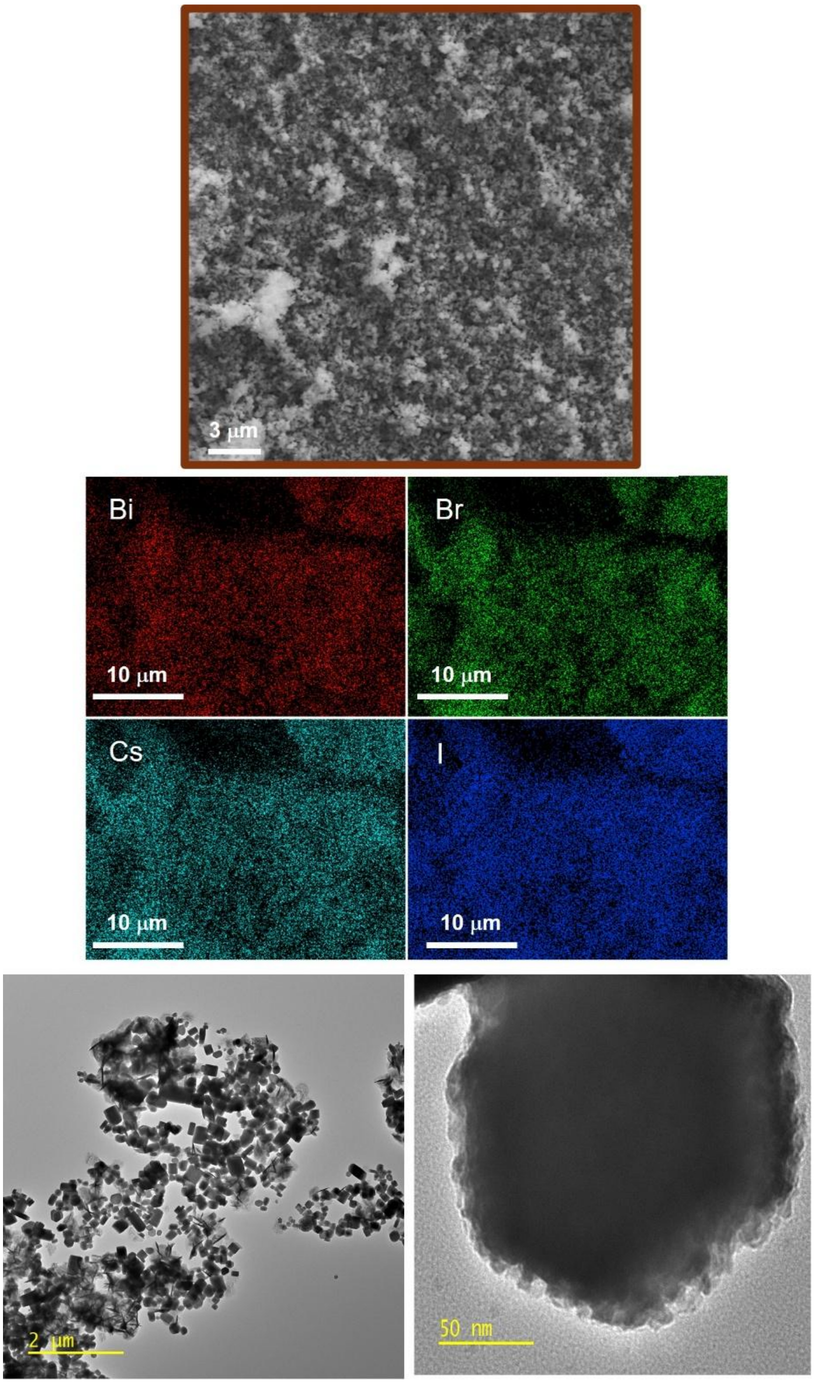

Figure 4S. SEM image, EDX mapping of the elements present in the $Cs_3Bi_2I_{4.5}Br_{4.5}$ material and additional TEM images.

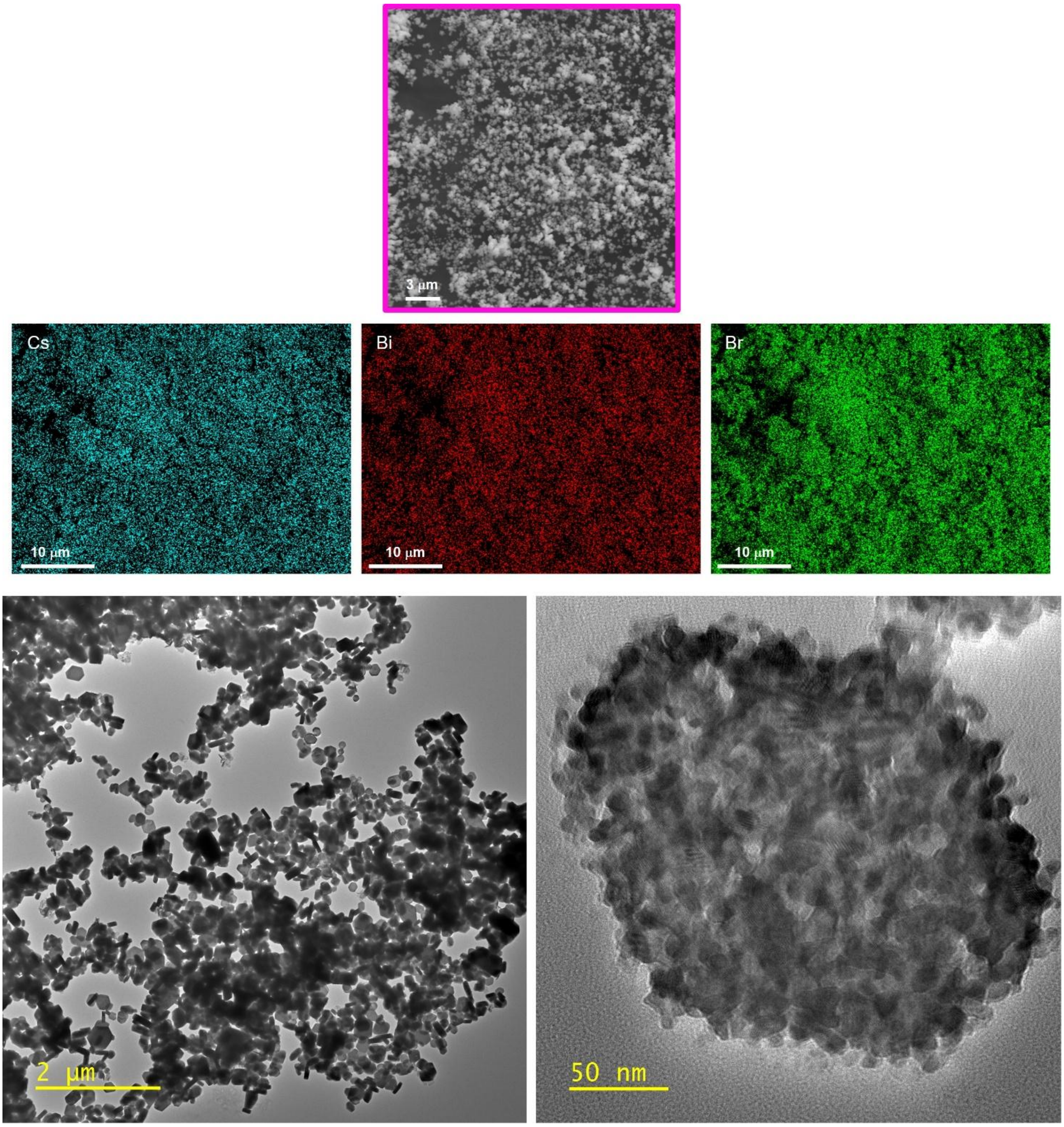

Figure 5S. SEM image, EDX mapping of the elements present in the L-$Cs_3Bi_2Br_9$ material and additional TEM images.

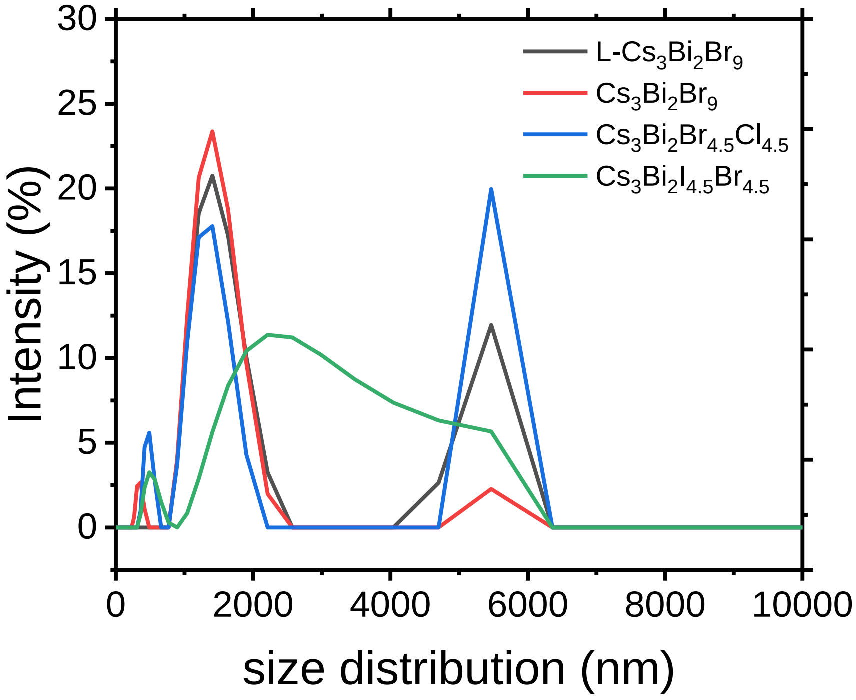

Figure 6S. DLS size distribution of the synthesized materials.

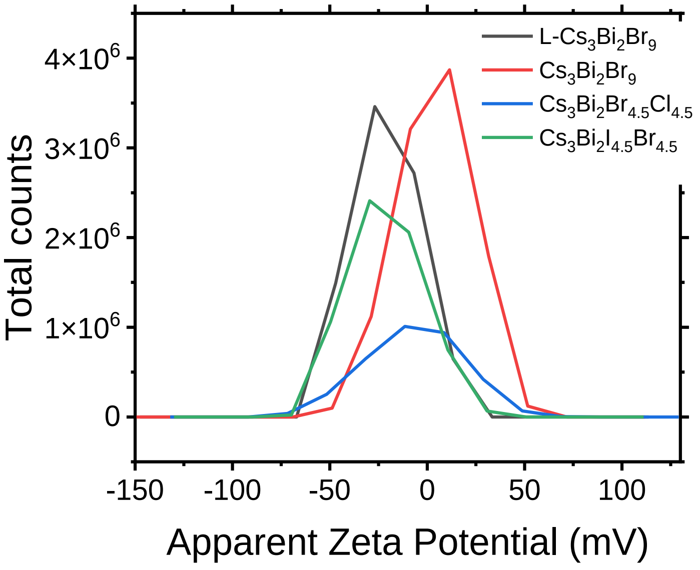

Figure 7S. Zeta potentials of the synthesized materials.

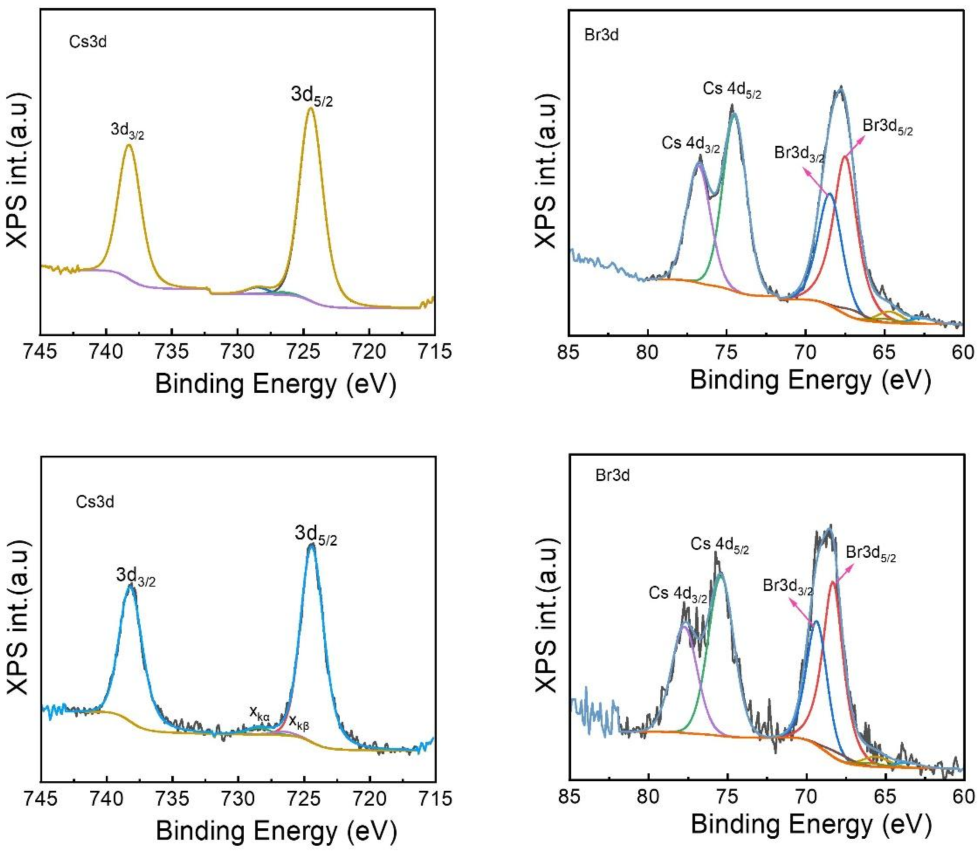

Figure 8S. XPS Cs 3d and Br 3d spectra of (top) conventional $Cs_3Bi_2Br_9$ and (down) L-$Cs_3Bi_2Br_9$ materials.

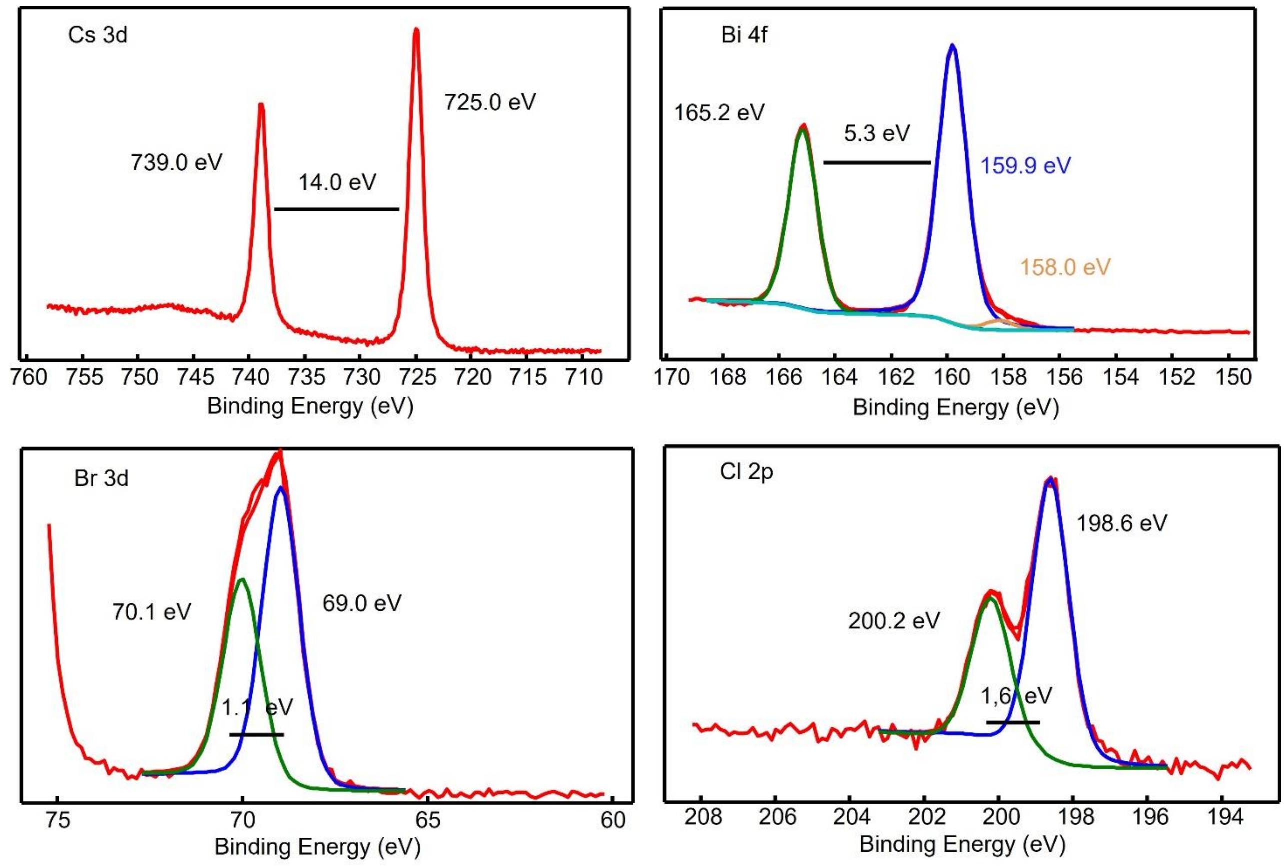

Figure 9S. XPS Cs 3d, Bi 4f, Br 3d and Cl 2p spectra of the conventional $Cs_3Bi_2Br_{4.5}Cl_{4.5}$ material.

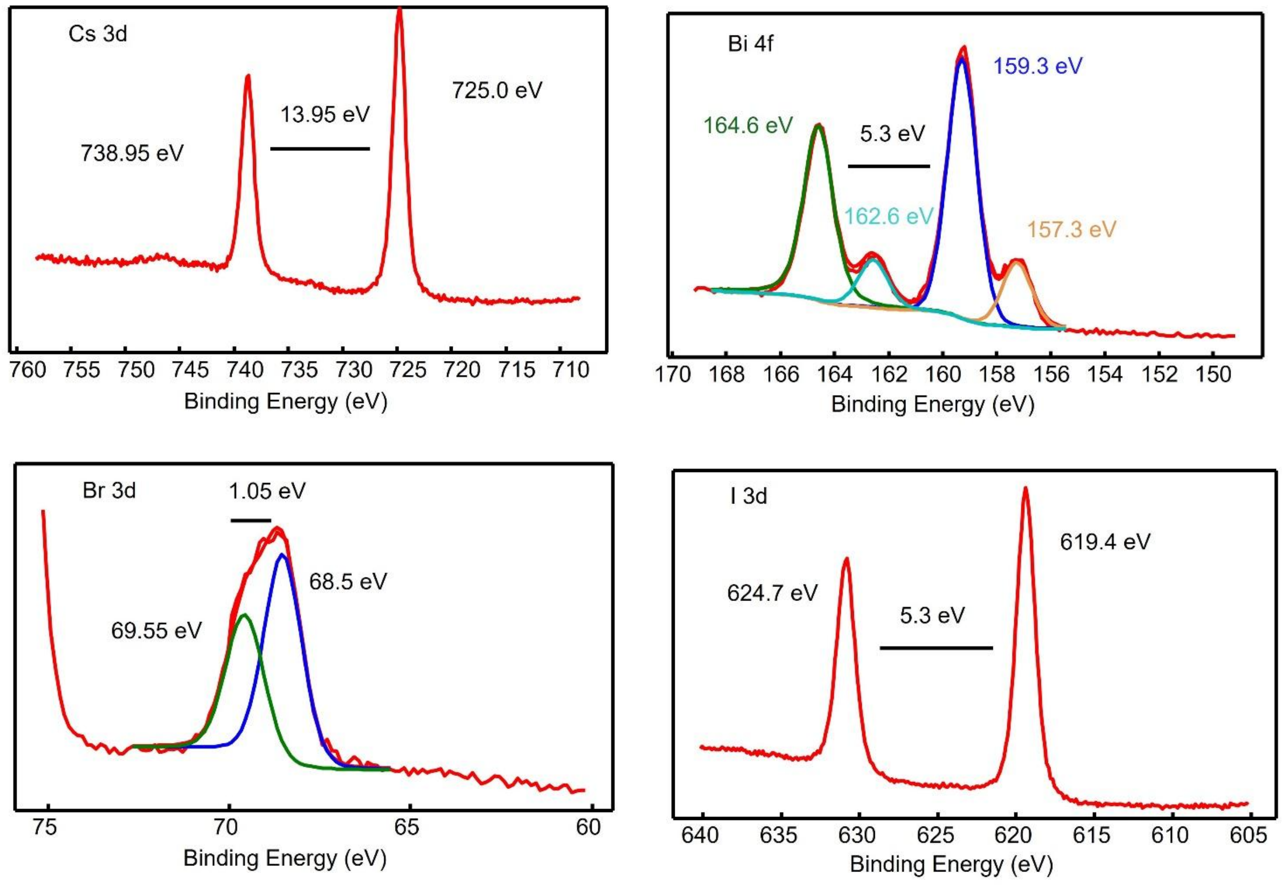

Figure 10S. XPS Cs 3d, Bi 4f, Br 3d and I 3d spectra of the conventional $Cs_3Bi_2I_{4.5}Br_{4.5}$ material.

***Ab-initio* structure determination by X-ray powder diffraction (XRPD)**

For the samples $Cs_3Bi_2Br_9$, L-$Cs_3Bi_2Br_9$, $Cs_3Bi_2Cl_{4.5}Br_{4.5}$ and $Cs_3Bi_2I_{4.5}Br_{4.5}$, all the steps of the *ab-initio* structure solution by XRPD data (*i.e.*, indexing, space group determination, integrated intensities extraction, structure solution and Rietveld refinement) were carried out by *EXPO* [S4]. In order to determine the true atomic composition of $Cs_3Bi_2Cl_{4.5}Br_{4.5}$ and $Cs_3Bi_2I_{4.5}Br_{4.5}$, their crystal structure was initially solved by *EXPO* assuming the atomic content of $Cs_3Bi_2Br_9$ (*i.e.*, by supposing that no substitution of Br atoms occurred); only at the final step, when the structure model was optimized by Rietveld refinement, the substitutional disorder for the two atomic sites occupied by Br atoms was taken into account.

The main results for $Cs_3Bi_2Br_9$, $Cs_3Bi_2Cl_{4.52}Br_{4.48}$ and $Cs_3Bi_2I_{4.56}Br_{4.44}$ are reported below; in the case of L-$Cs_3Bi_2Br_9$, being its crystal structure very similar to that one of $Cs_3Bi_2Br_9$, for the sake of brevity no details on the structure solution process are provided.

- ***Indexing***. The unit cell parameters were determined by *N-TREOR09* [S5], *i.e.*, the default indexing program implemented in *EXPO*, exploiting the information on 54, 77 and 40 experimental diffraction peaks for $Cs_3Bi_2Br_9$, $Cs_3Bi_2Cl_{4.5}Br_{4.5}$ and $Cs_3Bi_2I_{4.5}Br_{4.5}$, respectively. For all the compounds, the most plausible cell found by *N-TREOR09* was hexagonal [cell parameters (a=b, c) and cell volume (V): i) $Cs_3Bi_2Br_9$: a= 7.9561(3) Å, c= 9.8398(5) Å, V= 539.41(4) Å^3; ii) $Cs_3Bi_2Cl_{4.5}Br_{4.5}$: a= 7.967(4) Å, c= 9.5897(8) Å, V= 504.84(6) Å^3; $Cs_3Bi_2I_{4.5}Br_{4.5}$: a= 8.2403(6) Å, c= 10.1096(17) Å, V= 594.5(1) Å^3]; the confidence in the reliability of the hexagonal unit cells was supported by the large value of the M_{20} de Wolff figure of merit [S6], equal to 77, 98 and 43, respectively, and by the absence of unindexed lines.
- ***Space group determination***. The prior information on unit cell parameters and atomic content was exploited to determine the space group by performing:
  a) the integrated intensities extraction in *P*6/*mm* (*i.e.*, the space group with the largest Laue symmetry compatible with the geometry of the hexagonal unit cell and no extinction conditions);
  b) a statistical analysis on the suitably weighted integrated intensities, to find the systematic absences and calculate a probability value for each extinction symbol compatible with the trigonal-hexagonal crystal systems (*i.e.*, 12 extinction symbols, only hexagonal axes are taken into account) [S7-S8]. *EXPO* correctly identified the most probable extinction symbol (*i.e.*, *P* – – –) and one of the sixteen space groups referring to it (*i.e.*, *P*-3*m*1) was graphically selected to continue the *ab-initio* structure solution pathway.
- ***Full pattern decomposition***. The extraction of the integrated intensities of reflections was performed, based on the Le Bail algorithm [S9].

- ***Structure solution***

This step was carried out by applying Direct Methods (DM) [S10], that processed the integrated intensities and determined twenty different sets of phases of reflections; for each of them, *EXPO* performed a structure model optimization *via* an automatic procedure that provided, as final outcomes, twenty structure models ranked according to increasing values of R_F (*i.e.*, the agreement factor between observed and calculated structure factor moduli). Among them, the five-atoms structure model most crystallochemically plausible was graphically selected for the next and last step (*i.e.*, Rietveld refinement [S11]).

- ***Rietveld refinement***

The selected most promising model of $Cs_3Bi_2Br_9$, $Cs_3Bi_2Cl_{4.5}Br_{4.5}$ and $Cs_3Bi_2I_{4.5}Br_{4.5}$ was refined by Rietveld method. In the case of $Cs_3Bi_2Br_9$, the automatic Rietveld refinement, optimizing profile and structural parameters, was carried out. Figure 12SA shows the final Rietveld outcomes and the excellent agreement between observed (blue) and calculated (green) diffraction profiles, that are strongly overlapping and, consequently, characterized by very low R_p and R_{wp} agreement factors [S12] (see Table 1S). Similar matches were obtained at the end of the Rietveld refinement carried out for the two mixed-halides analogues of $Cs_3Bi_2Br_9$. For them, in the case of the two sites occupied by Br atoms, the substitutional disorder was taken into account by: 1) assuming that both the atomic sites were shared by the couple of halogens (I, Br), in the case of $Cs_3Bi_2I_{4.5}Br_{4.5}$, and by (Cl, Br) in the case of $Cs_3Bi_2Cl_{4.5}Br_{4.5}$; 2) assigning to the halogens of the site-sharing couples (I, Br) and (Cl, Br) the same starting site occupancy factor (*i.e.*, 0.5) and the same isotropic atomic displacement parameter, this last was constrained to be the same value during the Rietveld refinement. Furthermore, for the couples of atoms (I, Br) and (Cl, Br), the site occupancy factor was also refined by Rietveld method, in addition to the default refined parameters (*i.e.*, unit cell, background, full width at half maximum of the peak shape, peak asymmetry and zero-shift, fractional atomic coordinates and isotropic displacement parameters). At the end of the Rietveld refinement process, the final chemical formula (*FCF*) provided by *EXPO* for the two mixed-halides analogues of $Cs_3Bi_2Br_9$ was $Cs_3Bi_2Cl_{4.52}Br_{4.48}$ and $Cs_3Bi_2I_{4.56}Br_{4.44}$ (see Table 1 of the manuscript).

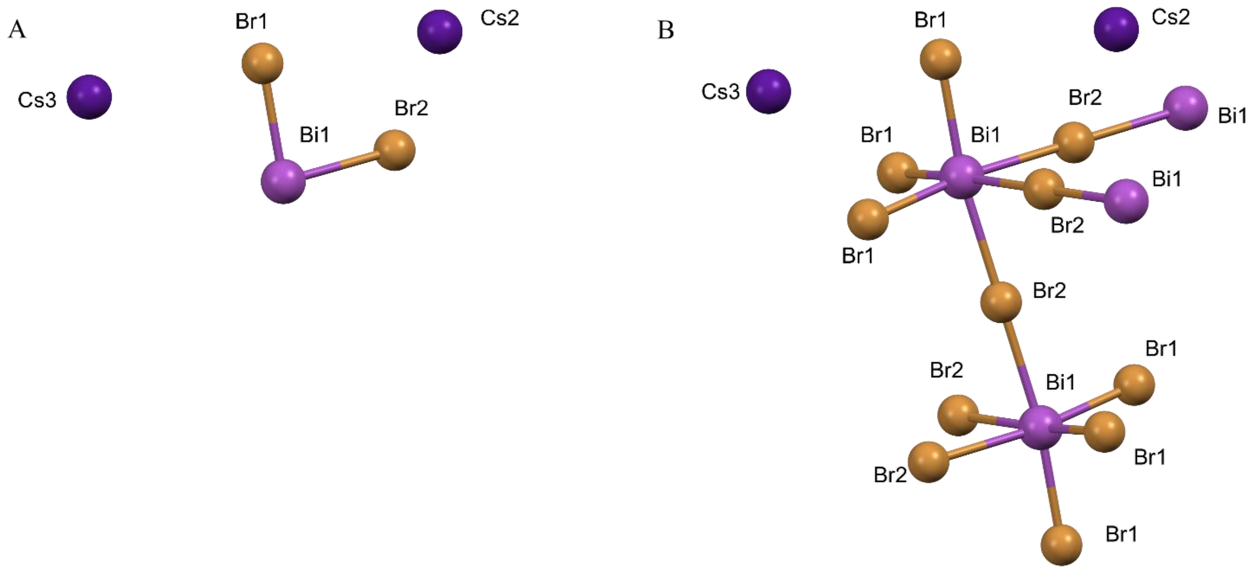

Figure 11S. $Cs_3Bi_2Br_9$: (A) View of the asymmetric unit and its local environment (B) showing the polyhedral coordination of Bi atoms.

A

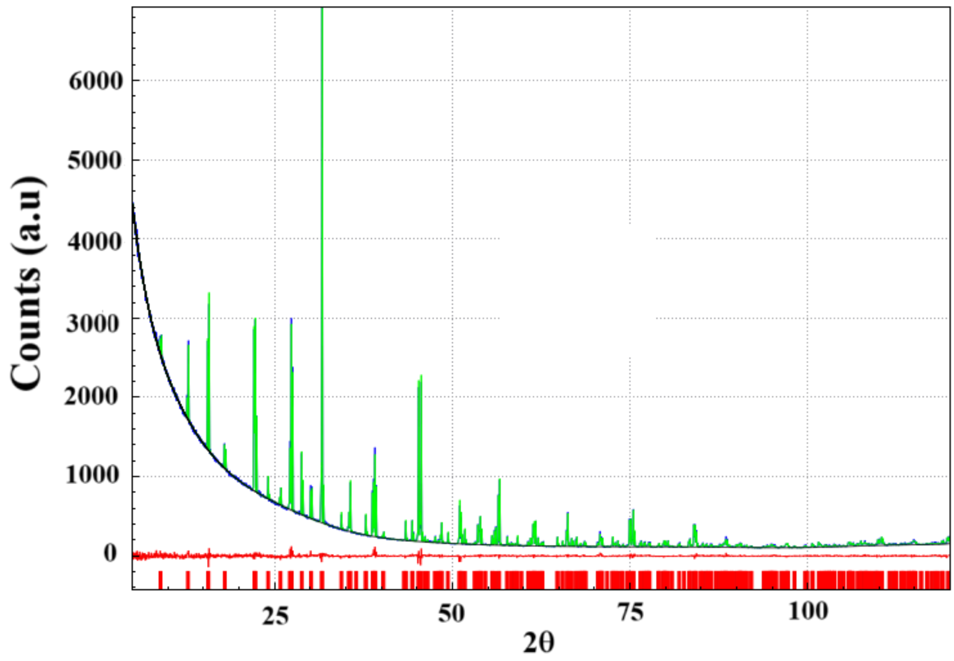

B

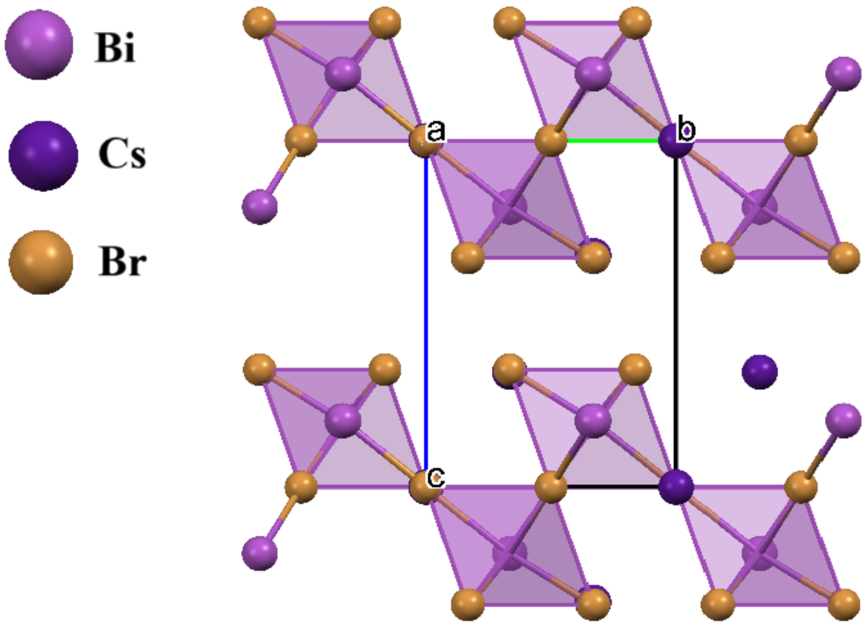

Figure 12S. $Cs_3Bi_2Br_9$: (A) Final Rietveld refinement results showing the calculated (green), observed (blue), difference (red) pattern and the calculated 2θ-position of reflections (red vertical bars at the bottom); the agreement factors R_p, and R_{wp} are 1.6% and 2.5% respectively. (B) a view of the crystal packing along *a*. Software used for molecular graphics: Mercury.[S13] Similar results were obtained for L-$Cs_3Bi_2Br_9$.

Table 1S. Crystal data, data collection and structure refinement details for $Cs_3Bi_2Br_9$, L-$Cs_3Bi_2Br_9$, $Cs_3Bi_2Cl_{4.52}Br_{4.48}$ and $Cs_3Bi_2I_{4.56}Br_{4.44}$.

Crystal data				
	**$Cs_3Bi_2Br_9$**	**L-$Cs_3Bi_2Br_9$**	**$Cs_3Bi_2Cl_{4.52}Br_{4.48}$**	**$Cs_3Bi_2I_{4.56}Br_{4.44}$**
M_r	1535.83	1535.83	1335.08	1749.56
Crystal system	Trigonal	Trigonal	Trigonal	Trigonal
Space group	*P*-3*m*1	*P*-3*m*1	*P*-3*m*1	*P*-3*m*1
Temperature (K)	293	293	293	293
a, *c* (Å)	7.95577(17),<br>9.8414(4)<br>539.45(3)	7.95544(17)<br>9.8399(4)<br>539.32(3)	7.7970(3)<br>9.5901(7)<br>504.90(4)	8.2405(15)<br>10.113(4)<br>594.7(3)
Data collection				
Diffractometer	Rigaku RINT2500 Powder X Ray Diffractometer			
Radiation type	Cu $K\alpha_1$			
Specimen mounting	Lindemann glass capillary			
Data collection mode	Transmission			
2θ values (°)	$2\theta_{min}$ = 5.00<br>$2\theta_{max}$ = 120.0<br>$2\theta_{step}$ = 0.02°	$2\theta_{min}$ = 5.00<br>$2\theta_{max}$ = 120.0<br>$2\theta_{step}$ = 0.02°	$2\theta_{min}$ = 10.00<br>$2\theta_{max}$ = 120.0<br>$2\theta_{step}$ = 0.02°	$2\theta_{min}$ = 10.0<br>$2\theta_{max}$ = 120.0<br>$2\theta_{step}$ = 0.02°
Rietveld Refinement				
R factors	R_p = 0.016<br>R_{wp} = 0.025<br>R_{Bragg} = 0.168	R_p = 0.018<br>R_{wp} = 0.029<br>R_{Bragg} = 0.090	R_p = 0.018<br>R_{wp} = 0.027<br>R_{Bragg} = 0.074	R_p = 0.019<br>R_{wp} = 0.029<br>R_{Bragg} = 0.140
Computer programs	*Applications*			
EXPO [S4]	Indexing, Space group determination, Structure solution (Direct Methods), Rietveld refinement			
Mercury [S13]	Molecular graphics			
CheckCIF [S14]	CIF validation			
publCIF [S15]	Preparation of material for publication			

Table 2S. $Cs_3Bi_2Br_9$: Fractional atomic coordinates and isotropic or equivalent isotropic displacement parameters (Å^2) and geometric parameters (Å, º).

	x	*y*	*z*	U_{iso}*/U_{eq}
Bi1	0.3333	0.6667	0.80822 (14)	0.0005 (3)*
Br1	0.1663	0.8337 (2)	0.6613 (3)	0.0256 (7)*
Br2	0.5000	1.0000	1.0000	0.0344 (14)*
Cs2	0.0000	1.0000	1.0000	0.0241 (12)*
Cs3	0.3333	0.6667	0.3319 (3)	0.0239 (6)*

Geometric parameters (Å, º)

Bi1—Br2	2.9727(9)	Cs3—Br1^{xi}	3.9785 (2)
Bi1—Br2^{i}	2.9727(9)	Cs3—Br1^{ii}	3.976 (3)
Bi1—Br2^{ii}	2.9727(9)	Br2—Bi1^{xii}	2.9727(9)
Bi1—Br1	2.7179 (19)	Br2—Cs3^{ix}	3.993(2)
Cs3—Br2^{iii}	3.993(2)	Br2—Cs3^{xiii}	3.993(2)
Cs3—Br2^{iv}	3.993(2)	Br2—Cs2^{xiv}	3.9779(2)
Cs3—Br2^{v}	3.993(2)	Br2—Cs2	3.9779(2)
Cs3—Br1^{vi}	3.9785 (2)	Br1—Cs3^{ix}	3.9785 (2)
Cs3—Br1^{vii}	3.9785 (2)	Br1—Cs3^{xi}	3.9785 (2)
Cs3—Br1^{i}	3.976 (3)	Cs2—Br2^{xv}	3.9779(2)
Cs3—Br1^{viii}	3.9785 (2)	Cs2—Br2^{xvi}	3.9779(2)
Cs3—Br1^{ix}	3.9785 (2)	Cs2—Br2^{xvii}	3.9779(2)
Cs3—Br1^{x}	3.9785 (2)	Cs2—Br2^{xviii}	3.9779(2)
Cs3—Br1	3.976 (3)	Cs2—Br2^{i}	3.9779(2)
Br2—Bi1—Br2^{i}	83.99	Br1^{vii}—Cs3—Br1^{ix}	59.85 (4)
Br2—Bi1—Br2^{ii}	83.99	Br1^{vii}—Cs3—Br1^{x}	119.97 (5)
Br2—Bi1—Br1	90.61 (5)	Br1^{vii}—Cs3—Br1	119.16 (5)
Bi1—Br2—Bi1^{xii}	179.98	Br1^{vii}—Cs3—Br1^{xi}	119.97 (5)
Bi1—Br2—Cs3^{ix}	85.70	Br1^{vii}—Cs3—Br1^{ii}	58.99 (4)
Bi1—Br2—Cs3^{xiii}	94.30	Br1^{i}—Cs3—Br1^{viii}	89.26 (5)
Bi1—Br2—Cs2^{xiv}	90.00 (1)	Br1^{i}—Cs3—Br1^{ix}	119.16 (5)
Bi1—Br2—Cs2	90.00 (1)	Br1^{i}—Cs3—Br1^{x}	119.16 (5)
Br2^{i}—Bi1—Br2^{ii}	83.99	Br1^{i}—Cs3—Br1	60.18 (4)
Br2^{i}—Bi1—Br1	90.61(5)	Br1^{i}—Cs3—Br1^{xi}	58.99 (4)
Br2^{ii}—Bi1—Br1	172.72	Br1^{i}—Cs3—Br1^{ii}	60.18 (4)
Bi1—Br1—Cs3	86.76 (5)	Br1^{viii}—Cs3—Br1^{ix}	119.97 (5)
Bi1—Br1—Cs3^{ix}	89.43 (5)	Br1^{viii}—Cs3—Br1^{x}	59.85 (4)
Bi1—Br1—Cs3^{xi}	89.43 (5)	Br1^{viii}—Cs3—Br1	58.99 (4)
Br2^{iii}—Cs3—Br2^{iv}	59.75	Br1^{viii}—Cs3—Br1^{xi}	60.13 (4)
Br2^{iii}—Cs3—Br2^{v}	59.75	Br1^{viii}—Cs3—Br1^{ii}	119.16 (5)
Br2^{iii}—Cs3—Br1^{vi}	61.05	Br1^{ix}—Cs3—Br1^{x}	60.13 (4)

Br2^{iii}—Cs3—Br1^{vii}	61.05	Br1^{ix}—Cs3—Br1	89.26 (5)
Br2^{iii}—Cs3—Br1^{i}	120.04	Br1^{ix}—Cs3—Br1^{xi}	178.08 (6)
Br2^{iii}—Cs3—Br1^{viii}	120.80	Br1^{ix}—Cs3—Br1^{ii}	58.99 (4)
Br2^{iii}—Cs3—Br1^{ix}	90.74	Br1^{x}—Cs3—Br1	58.99 (4)
Br2^{iii}—Cs3—Br1^{x}	120.80	Br1^{x}—Cs3—Br1^{xi}	119.97 (5)
Br2^{iii}—Cs3—Br1	179.74	Br1^{x}—Cs3—Br1^{ii}	89.26 (5)
Br2^{iii}—Cs3—Br1^{xi}	90.74	Br1—Cs3—Br1^{xi}	89.26 (5)
Br2^{iii}—Cs3—Br1^{ii}	120.04	Br1—Cs3—Br1^{ii}	60.18 (4)
Br2^{iv}—Cs3—Br2^{v}	59.75	Cs3—Br1—Cs3^{ix}	90.74 (5)
Br2^{iv}—Cs3—Br1^{vi}	90.74	Cs3—Br1—Cs3^{xi}	90.74 (5)
Br2^{iv}—Cs3—Br1^{vii}	120.80	Br1^{xi}—Cs3—Br1^{ii}	119.16 (5)
Br2^{iv}—Cs3—Br1^{i}	120.04	Bi1^{xii}—Br2—Cs3^{ix}	94.30
Br2^{iv}—Cs3—Br1^{viii}	61.05	Bi1^{xii}—Br2—Cs3^{xiii}	85.70
Br2^{iv}—Cs3—Br1^{ix}	120.80	Bi1^{xii}—Br2—Cs2^{xiv}	90.00
Br2^{iv}—Cs3—Br1^{x}	90.74	Bi1^{xii}—Br2—Cs2	90.00
Br2^{iv}—Cs3—Br1	120.04	Cs3^{ix}—Br2—Cs3^{xiii}	179.97
Br2^{iv}—Cs3—Br1^{xi}	61.05	Cs3^{ix}—Br2—Cs2^{xiv}	90.00
Br2^{iv}—Cs3—Br1^{ii}	179.74	Cs3^{ix}—Br2—Cs2	90.00
Br2^{v}—Cs3—Br1^{vi}	120.80	Cs3^{xiii}—Br2—Cs2^{xiv}	90.00
Br2^{v}—Cs3—Br1^{vii}	90.74	Cs3^{xiii}—Br2—Cs2	90.00
Br2^{v}—Cs3—Br1^{i}	179.74	Cs2^{xiv}—Br2—Cs2	179.97
Br2^{v}—Cs3—Br1^{viii}	90.74	Br2—Cs2—Br2^{xv}	60.00
Br2^{v}—Cs3—Br1^{ix}	61.05	Br2—Cs2—Br2^{xvi}	120.00
Br2^{v}—Cs3—Br1^{x}	61.05	Br2—Cs2—Br2^{xvii}	120.00
Br2^{v}—Cs3—Br1	120.04	Br2—Cs2—Br2^{xviii}	179.97
Br2^{v}—Cs3—Br1^{xi}	120.80	Br2—Cs2—Br2^{i}	60.00
Br2^{v}—Cs3—Br1^{ii}	120.04	Cs3^{ix}—Br1—Cs3^{xi}	178.08 (6)
Br1^{vi}—Cs3—Br1^{vii}	60.13 (4)	Br2^{xv}—Cs2—Br2^{xvi}	179.97
Br1^{vi}—Cs3—Br1^{i}	58.99 (4)	Br2^{xv}—Cs2—Br2^{xvii}	60.00
Br1^{vi}—Cs3—Br1^{viii}	119.97 (5)	Br2^{xv}—Cs2—Br2^{xviii}	120.00
Br1^{vi}—Cs3—Br1^{ix}	119.97 (5)	Br2^{xv}—Cs2—Br2^{i}	120.00
Br1^{vi}—Cs3—Br1^{x}	178.08 (6)	Br2^{xvi}—Cs2—Br2^{xvii}	120.00
Br1^{vi}—Cs3—Br1	119.16 (5)	Br2^{xvi}—Cs2—Br2^{xviii}	60.00
Br1^{vi}—Cs3—Br1^{xi}	59.85 (4)	Br2^{xvi}—Cs2—Br2^{i}	60.00
Br1^{vi}—Cs3—Br1^{ii}	89.26 (5)	Br2^{xvii}—Cs2—Br2^{xviii}	60.00
Br1^{vii}—Cs3—Br1^{i}	89.26 (5)	Br2^{xvii}—Cs2—Br2^{i}	179.97
Br1^{vii}—Cs3—Br1^{viii}	178.08 (6)	Br2^{xviii}—Cs2—Br2^{i}	120.00

Symmetry codes: (i) -*y*+1, *x*-*y*+1, *z*; (ii) -*x*+*y*, -*x*+1, *z*; (iii) -*x*+*y*, -*x*+1, *z*-1; (iv) -*y*+1, *x*-*y*+1, *z*-1; (v) *x*, *y*, *z*-1; (vi) *x*-*y*+1, -*y*+1, -*z*+1; (vii) -*x*+1, -*x*+*y*, -*z*+1; (viii) -*x*, -*x*+*y*, -*z*+1; (ix) *y*, *x*+1, -*z*+1; (x) *x*-*y*+1, -*y*+2, -*z*+1; (xi) *y*-1, *x*, -*z*+1; (xii) *y*, *x*+1, -*z*+2; (xiii) *x*, *y*, *z*+1; (xiv) *x*+1, *y*, *z*; (xv) -*x*+*y*, -*x*+2, *z*; (xvi) -*x*+*y*-1, -*x*+1, *z*; (xvii) -*y*+1, *x*-*y*+2, *z*; (xviii) *x*-1, *y*, *z*.

Table 3S. $Cs_3Bi_2Cl_{4.52}Br_{4.48}$: Fractional atomic coordinates, isotropic or equivalent isotropic displacement parameters (Å^2) and site occupancy factor (*SOF*) and geometric parameters (Å, º)

	x	*y*	*z*	U_{iso}*/U_{eq}	*SOF*
Bi1	0.3333	0.6667	0.8175 (2)	0.0000 (4)*	1.0
Br1	0.6666	0.8333 (5)	0.6654 (5)	0.0093*	0.640 (7)
Cl1	0.6666	0.8333	0.6654	0.0093 (7)*	0.360 (7)
Br2	0.5000	1.0000	1.0000	0.052 (3)*	0.214 (17)
Cl2	0.5000	1.0000	1.0000	0.0518*	0.786 (17)
Cs2	1.0000	1.0000	1.0000	0.0053 (12)*	1.0
Cs3	0.6667	0.3333	0.6694 (4)	0.0108 (6)*	1.0

Geometric parameters (Å, º)

Bi1—Cl1	2.6818	Cs2—Br1^{x}	3.9195
Bi1—Cl1^{i}	2.6818	Cs2—Br1^{xi}	3.9195
Bi1—Cl1^{ii}	2.6818	Cs2—Br1^{xii}	3.9196
Bi1—Cl2^{i}	2.8514	Cs2—Br1^{xiii}	3.9196
Bi1—Cl2^{ii}	2.8514	Cs2—Br1^{viii}	3.9196
Bi1—Cl2	2.8514	Cs2—Br1	3.9195
Cs3—Br1^{iii}	3.899 (4)	Cs2—Br2^{xiv}	3.8985
Cs3—Br1^{i}	3.899 (2)	Cs2—Br2	3.8985
Cs3—Br1^{iv}	3.921 (5)	Cs2—Br2^{viii}	3.8985
Cs3—Br1	3.899 (4)	Cs2—Br2^{xv}	3.8985
Cs3—Br1^{v}	3.921 (5)	Cs2—Br2^{i}	3.8985
Cs3—Br1^{vi}	3.899 (2)	Cs2—Br2^{xii}	3.8985
Cs3—Br1^{vii}	3.899 (2)	Br1—Cs3^{iv}	3.921 (5)
Cs3—Br1^{viii}	3.899 (2)	Br1—Cs3^{xvi}	3.899 (4)
Cs3—Br1^{ix}	3.921 (5)	Cl2—Bi1^{xvii}	2.8514
Cs3—Br2^{i}	3.8884	Br2—Cs3^{xiii}	3.8884
Cs3—Br2^{viii}	3.8884	Br2—Cs3^{xvi}	3.8884
Cs3—Br2^{iii}	3.8884	Br2—Cs2^{xviii}	3.8985
Cl1—Bi1—Cl1^{i}	93.24	Br1^{ix}—Cs3—Br2^{viii}	120.10
Cl1—Bi1—Cl1^{ii}	93.24	Br1^{ix}—Cs3—Br2^{iii}	120.10
Cl1—Bi1—Cl2^{i}	90.15	Br2^{i}—Cs3—Br2^{viii}	60.17
Cl1—Bi1—Cl2^{ii}	175.07	Br2^{i}—Cs3—Br2^{iii}	60.17
Cl1—Bi1—Cl2	90.15	Br2^{viii}—Cs3—Br2^{iii}	60.17
Cl1^{i}—Bi1—Cl1^{ii}	93.24	Br1^{x}—Cs2—Br1^{xi}	59.65
Cl1^{i}—Bi1—Cl2^{i}	90.15	Br1^{x}—Cs2—Br1^{xii}	120.35
Cl1^{i}—Bi1—Cl2^{ii}	90.15	Br1^{x}—Cs2—Br1^{xiii}	59.65
Cl1^{i}—Bi1—Cl2	175.07	Br1^{x}—Cs2—Br1^{viii}	180.00
Cl1^{ii}—Bi1—Cl2^{i}	175.06	Br1^{x}—Cs2—Br1	120.35
Cl1^{ii}—Bi1—Cl2^{ii}	90.15	Br1^{x}—Cs2—Br2^{xiv}	119.82

Cl1^{ii}—Bi1—Cl2	90.15	Br1^{x}—Cs2—Br2	60.18
Cl2^{i}—Bi1—Cl2^{ii}	86.25	Br1^{x}—Cs2—Br2^{viii}	119.82
Cl2^{i}—Bi1—Cl2	86.25	Br1^{x}—Cs2—Br2^{xv}	60.18
Cl2^{ii}—Bi1—Cl2	86.25	Br1^{x}—Cs2—Br2^{i}	90.00
Bi1—Cl2—Bi1^{xvii}	179.97	Br1^{x}—Cs2—Br2^{xii}	90.00
Br1^{iii}—Cs3—Br1^{i}	119.99 (9)	Br1^{xi}—Cs2—Br1^{xii}	120.35
Br1^{iii}—Cs3—Br1^{iv}	119.28 (9)	Br1^{xi}—Cs2—Br1^{xiii}	59.65
Br1^{iii}—Cs3—Br1	178.88 (10)	Br1^{xi}—Cs2—Br1^{viii}	120.35
Br1^{iii}—Cs3—Br1^{v}	89.54 (9)	Br1^{xi}—Cs2—Br1	180.00
Br1^{iii}—Cs3—Br1^{vi}	60.00 (8)	Br1^{xi}—Cs2—Br2^{xiv}	60.18
Br1^{iii}—Cs3—Br1^{vii}	59.99 (8)	Br1^{xi}—Cs2—Br2	119.82
Br1^{iii}—Cs3—Br1^{viii}	119.99 (9)	Br1^{xi}—Cs2—Br2^{viii}	90.00
Br1^{iii}—Cs3—Br1^{ix}	59.66 (8)	Br1^{xi}—Cs2—Br2^{xv}	90.00
Br1^{iii}—Cs3—Br2^{i}	120.61	Br1^{xi}—Cs2—Br2^{i}	119.82
Br1^{iii}—Cs3—Br2^{viii}	90.46	Br1^{xi}—Cs2—Br2^{xii}	60.18
Br1^{iii}—Cs3—Br2^{iii}	60.44	Br1^{xii}—Cs2—Br1^{xiii}	179.98
Br1^{i}—Cs3—Br1^{iv}	89.54 (9)	Br1^{xii}—Cs2—Br1^{viii}	59.65
Br1^{i}—Cs3—Br1	59.99 (8)	Br1^{xii}—Cs2—Br1	59.65
Br1^{i}—Cs3—Br1^{v}	59.66 (8)	Br1^{xii}—Cs2—Br2^{xiv}	90.00
Br1^{i}—Cs3—Br1^{vi}	178.88 (10)	Br1^{xii}—Cs2—Br2	90.00
Br1^{i}—Cs3—Br1^{vii}	60.00 (8)	Br1^{xii}—Cs2—Br2^{viii}	119.82
Br1^{i}—Cs3—Br1^{viii}	119.99 (9)	Br1^{xii}—Cs2—Br2^{xv}	60.18
Br1^{i}—Cs3—Br1^{ix}	119.28 (9)	Br1^{xii}—Cs2—Br2^{i}	119.82
Br1^{i}—Cs3—Br2^{i}	60.44	Br1^{xii}—Cs2—Br2^{xii}	60.18
Br1^{i}—Cs3—Br2^{viii}	120.61	Br1^{xiii}—Cs2—Br1^{viii}	120.35
Br1^{i}—Cs3—Br2^{iii}	90.46	Br1^{xiii}—Cs2—Br1	120.35
Br1^{iv}—Cs3—Br1	59.66 (8)	Br1^{xiii}—Cs2—Br2^{xiv}	90.00
Br1^{iv}—Cs3—Br1^{v}	59.62 (8)	Br1^{xiii}—Cs2—Br2	90.00
Br1^{iv}—Cs3—Br1^{vi}	89.54 (9)	Br1^{xiii}—Cs2—Br2^{viii}	60.18
Br1^{iv}—Cs3—Br1^{vii}	119.28 (9)	Br1^{xiii}—Cs2—Br2^{xv}	119.82
Br1^{iv}—Cs3—Br1^{viii}	59.66 (8)	Br1^{xiii}—Cs2—Br2^{i}	60.18
Br1^{iv}—Cs3—Br1^{ix}	59.62 (8)	Br1^{xiii}—Cs2—Br2^{xii}	119.82
Br1^{iv}—Cs3—Br2^{i}	120.10	Br1^{viii}—Cs2—Br1	59.65
Br1^{iv}—Cs3—Br2^{viii}	120.10	Br1^{viii}—Cs2—Br2^{xiv}	60.18
Br1^{iv}—Cs3—Br2^{iii}	179.66	Br1^{viii}—Cs2—Br2	119.82
Br1—Cs3—Br1^{v}	89.54 (9)	Br1^{viii}—Cs2—Br2^{viii}	60.18
Br1—Cs3—Br1^{vi}	119.99 (9)	Br1^{viii}—Cs2—Br2^{xv}	119.82
Br1—Cs3—Br1^{vii}	119.99 (9)	Br1^{viii}—Cs2—Br2^{i}	90.00
Br1—Cs3—Br1^{viii}	60.00 (8)	Br1^{viii}—Cs2—Br2^{xii}	90.00
Br1—Cs3—Br1^{ix}	119.28 (9)	Br1—Cs2—Br2^{xiv}	119.82
Br1—Cs3—Br2^{i}	60.44	Br1—Cs2—Br2	60.18
Br1—Cs3—Br2^{viii}	90.46	Br1—Cs2—Br2^{viii}	90.00
Br1—Cs3—Br2^{iii}	120.61	Br1—Cs2—Br2^{xv}	90.00

Cs3—Br1—Cs2	89.54	Br1—Cs2—Br2^{i}	60.18
Cs3—Br1—Cs3^{iv}	90.46 (9)	Br1—Cs2—Br2^{xii}	119.82
Cs3—Br1—Cs3^{xvi}	178.88 (12)	Cs2—Br1—Cs3^{iv}	179.97
Br1^{v}—Cs3—Br1^{vi}	119.28 (9)	Cs2—Br1—Cs3^{xvi}	89.54
Br1^{v}—Cs3—Br1^{vii}	59.66 (8)	Br2^{xiv}—Cs2—Br2	179.97
Br1^{v}—Cs3—Br1^{viii}	119.28 (9)	Br2^{xiv}—Cs2—Br2^{viii}	60.00
Br1^{v}—Cs3—Br1^{ix}	59.62 (8)	Br2^{xiv}—Cs2—Br2^{xv}	120.00
Br1^{v}—Cs3—Br2^{i}	120.10	Br2^{xiv}—Cs2—Br2^{i}	120.00
Br1^{v}—Cs3—Br2^{viii}	179.66	Br2^{xiv}—Cs2—Br2^{xii}	60.00
Br1^{v}—Cs3—Br2^{iii}	120.10	Br2—Cs2—Br2^{viii}	120.00
Br1^{vi}—Cs3—Br1^{vii}	119.99 (9)	Br2—Cs2—Br2^{xv}	60.00
Br1^{vi}—Cs3—Br1^{viii}	59.99 (8)	Br2—Cs2—Br2^{i}	60.00
Br1^{vi}—Cs3—Br1^{ix}	59.66 (8)	Br2—Cs2—Br2^{xii}	120.00
Br1^{vi}—Cs3—Br2^{i}	120.61	Cs2—Br2—Cs3^{xiii}	90.00
Br1^{vi}—Cs3—Br2^{viii}	60.44	Cs2—Br2—Cs3^{xvi}	90.00
Br1^{vi}—Cs3—Br2^{iii}	90.46	Cs2—Br2—Cs2^{xviii}	179.97
Br1^{vii}—Cs3—Br1^{viii}	178.88 (10)	Br2^{viii}—Cs2—Br2^{xv}	179.97
Br1^{vii}—Cs3—Br1^{ix}	89.54 (9)	Br2^{viii}—Cs2—Br2^{i}	60.00
Br1^{vii}—Cs3—Br2^{i}	90.46	Br2^{viii}—Cs2—Br2^{xii}	120.00
Br1^{vii}—Cs3—Br2^{viii}	120.61	Br2^{xv}—Cs2—Br2^{i}	120.00
Br1^{vii}—Cs3—Br2^{iii}	60.44	Br2^{xv}—Cs2—Br2^{xii}	60.00
Br1^{viii}—Cs3—Br1^{ix}	89.54 (9)	Br2^{i}—Cs2—Br2^{xii}	180.00
Br1^{viii}—Cs3—Br2^{i}	90.46	Cs3^{iv}—Br1—Cs3^{xvi}	90.46 (9)
Br1^{viii}—Cs3—Br2^{viii}	60.44	Cs3^{xiii}—Br2—Cs3^{xvi}	179.97
Br1^{viii}—Cs3—Br2^{iii}	120.61	Cs3^{xiii}—Br2—Cs2^{xviii}	90.00
Br1^{ix}—Cs3—Br2^{i}	179.66	Cs3^{xvi}—Br2—Cs2^{xviii}	90.00

Symmetry codes: (i) -*x*+*y*, -*x*+1, *z*; (ii) -*y*+1, *x*-*y*+1, *z*; (iii) *x*, *y*-1, *z*; (iv) *y*, *x*, -*z*+1; (v) -*x*+1, -*x*+*y*, -*z*+1; (vi) -*x*+*y*+1, -*x*+1, *z*; (vii) -*y*+1, *x*-*y*, *z*; (viii) -*y*+2, *x*-*y*+1, *z*; (ix) *x*-*y*+1, -*y*+1, -*z*+1; (x) *x*-*y*+1, -*y*+2, -*z*+2; (xi) -*x*+2, -*x*+*y*+1, -*z*+2; (xii) -*x*+*y*+1, -*x*+2, *z*; (xiii) *y*, *x*, -*z*+2; (xiv) *x*+1, *y*, *z*; (xv) -*y*+2, *x*-*y*+2, *z*; (xvi) *x*, *y*+1, *z*; (xvii) *y*, *x*+1, -*z*+2; (xviii) *x*-1, *y*, *z*.

Table 4S. $Cs_3Bi_2I_{4.56}Br_{4.44}$: Fractional atomic coordinates, isotropic or equivalent isotropic displacement parameters (Å^2) and site occupancy factor (*SOF*) and geometric parameters (Å, º)

	x	*y*	*z*	U_{iso}*/U_{eq}	*SOF*
Bi1	0.6667	0.3333	0.1776 (6)	0.0000 (12)*	1.0
Br1	0.3250	0.1625	0.3332	0.0000 (15)*	0.48 (2)
I1	0.3250	0.1625 (10)	0.3332 (9)	0.0000*	0.52 (2)
Br2	0.5000	0.0000	0.0000	0.075 (4)*	0.52 (5)
I2	0.5000	0.0000	0.0000	0.0753*	0.48 (5)
Cs2	0.0000	0.0000	0.0000	0.000 (4)*	1.0
Cs3	0.3333	-0.3333	0.3096 (11)	0.004 (2)*	1.0

Geometric parameters (Å, º)

Cs2—I1	4.0902	I1—Cs3^{ix}	4.128 (8)
Cs2—I1^{i}	4.0902	I1—Cs3	4.128 (8)
Cs2—I1^{ii}	4.0902	Bi1—Br1^{x}	2.9018
Cs2—I1^{iii}	4.0902	Bi1—Br1^{vi}	2.9017
Cs2—I1^{iv}	4.0902	Bi1—I2	2.9809
Cs2—I1^{v}	4.0902	I2—Cs2^{xi}	4.1202
Cs2—I2^{vi}	4.1202	I2—Cs3	3.9322
Cs2—I2^{v}	4.1202	Cs3—I1^{xii}	4.128 (4)
Cs2—I2^{ii}	4.1202	Cs3—I1^{x}	4.128 (4)
Cs2—I2^{vii}	4.1202	Cs3—I1^{vii}	4.128 (4)
Cs2—I2^{viii}	4.1202	Cs3—I1^{xiii}	4.128 (8)
Cs2—I2	4.1202	Cs3—I1^{v}	4.128 (4)

I1—Cs2—I1^{i}	121.18	I1^{v}—Cs2—I2^{vii}	60.59
I1—Cs2—I1^{ii}	58.82	I1^{v}—Cs2—I2^{viii}	90.00
I1—Cs2—I1^{iii}	121.18	I1^{v}—Cs2—I2	90.00
I1—Cs2—I1^{iv}	179.98	I2^{vi}—Cs2—I2^{v}	180.00
I1—Cs2—I1^{v}	58.82	I2^{vi}—Cs2—I2^{ii}	60.00
I1—Cs2—I2^{vi}	60.59	I2^{vi}—Cs2—I2^{vii}	120.00
I1—Cs2—I2^{v}	119.41	I2^{vi}—Cs2—I2^{viii}	120.00
I1—Cs2—I2^{ii}	90.00	I2^{vi}—Cs2—I2	60.00
I1—Cs2—I2^{vii}	90.00	I2^{v}—Cs2—I2^{ii}	120.00
I1—Cs2—I2^{viii}	119.41	I2^{v}—Cs2—I2^{vii}	60.00
I1—Cs2—I2	60.59	I2^{v}—Cs2—I2^{viii}	60.00
Cs2—I1—Cs3^{ix}	87.75	I2^{v}—Cs2—I2	120.00
Cs2—I1—Cs3	87.75	I2^{ii}—Cs2—I2^{vii}	179.98
I1^{i}—Cs2—I1^{ii}	121.18	I2^{ii}—Cs2—I2^{viii}	60.00
I1^{i}—Cs2—I1^{iii}	58.82	I2^{ii}—Cs2—I2	120.00
I1^{i}—Cs2—I1^{iv}	58.82	I2^{vii}—Cs2—I2^{viii}	120.00
I1^{i}—Cs2—I1^{v}	180.00	I2^{vii}—Cs2—I2	60.00
I1^{i}—Cs2—I2^{vi}	60.59	I2^{viii}—Cs2—I2	179.98

I1^{i}—Cs2—I2^{v}	119.41	Cs2—I2—Bi1	90.00
I1^{i}—Cs2—I2^{ii}	60.59	Cs2—I2—Cs2^{xi}	179.98
I1^{i}—Cs2—I2^{vii}	119.41	Cs2—I2—Cs3	90.00
I1^{i}—Cs2—I2^{viii}	90.00	Cs3^{ix}—I1—Cs3	173.2 (2)
I1^{i}—Cs2—I2	90.00	I1—Cs3—I2	61.79
I1^{ii}—Cs2—I1^{iii}	180.00	I1—Cs3—I1^{xii}	119.7 (2)
I1^{ii}—Cs2—I1^{iv}	121.18	I1—Cs3—I1^{x}	61.55 (16)
I1^{ii}—Cs2—I1^{v}	58.82	I1—Cs3—I1^{vii}	119.7 (2)
I1^{ii}—Cs2—I2^{vi}	90.00	I1—Cs3—I1^{xiii}	173.2 (2)
I1^{ii}—Cs2—I2^{v}	90.00	I1—Cs3—I1^{v}	58.23 (16)
I1^{ii}—Cs2—I2^{ii}	60.59	Br1^{x}—Bi1—Br1^{vi}	93.40
I1^{ii}—Cs2—I2^{vii}	119.41	Br1^{x}—Bi1—I2	89.50
I1^{ii}—Cs2—I2^{viii}	60.59	Br1^{vi}—Bi1—I2	175.76
I1^{ii}—Cs2—I2	119.41	Bi1—I2—Cs2^{xi}	90.00
I1^{iii}—Cs2—I1^{iv}	58.82	Bi1—I2—Cs3	90.17
I1^{iii}—Cs2—I1^{v}	121.18	Cs2^{xi}—I2—Cs3	90.00
I1^{iii}—Cs2—I2^{vi}	90.00	I2—Cs3—I1^{xii}	92.13
I1^{iii}—Cs2—I2^{v}	90.00	I2—Cs3—I1^{x}	61.79
I1^{iii}—Cs2—I2^{ii}	119.41	I2—Cs3—I1^{vii}	124.98
I1^{iii}—Cs2—I2^{vii}	60.59	I2—Cs3—I1^{xiii}	124.98
I1^{iii}—Cs2—I2^{viii}	119.41	I2—Cs3—I1^{v}	92.13
I1^{iii}—Cs2—I2	60.59	I1^{xii}—Cs3—I1^{x}	58.23 (16)
I1^{iv}—Cs2—I1^{v}	121.18	I1^{xii}—Cs3—I1^{vii}	119.7 (2)
I1^{iv}—Cs2—I2^{vi}	119.41	I1^{xii}—Cs3—I1^{xiii}	61.55 (16)
I1^{iv}—Cs2—I2^{v}	60.59	I1^{xii}—Cs3—I1^{v}	173.2 (2)
I1^{iv}—Cs2—I2^{ii}	90.00	I1^{x}—Cs3—I1^{vii}	173.2 (2)
I1^{iv}—Cs2—I2^{vii}	90.00	I1^{x}—Cs3—I1^{xiii}	119.7 (2)
I1^{iv}—Cs2—I2^{viii}	60.59	I1^{x}—Cs3—I1^{v}	119.7 (2)
I1^{iv}—Cs2—I2	119.41	I1^{vii}—Cs3—I1^{xiii}	58.23 (16)
I1^{v}—Cs2—I2^{vi}	119.41	I1^{vii}—Cs3—I1^{v}	61.55 (16)
I1^{v}—Cs2—I2^{v}	60.59	I1^{xiii}—Cs3—I1^{v}	119.7 (2)
I1^{v}—Cs2—I2^{ii}	119.41		

Symmetry codes: (i) *y*, *x*, -*z*; (ii) -*y*, *x*-*y*, *z*; (iii) *x*-*y*, -*y*, -*z*; (iv) -*x*, -*x*+*y*, -*z*; (v) -*x*+*y*, -*x*, *z*; (vi) -*x*+*y*+1, -*x*+1, *z*; (vii) -*y*, *x*-*y*-1, *z*; (viii) *x*-1, *y*, *z*; (ix) *x*, *y*+1, *z*; (x) -*y*+1, *x*-*y*, *z*; (xi) *x*+1, *y*, *z*; (xii) -*x*+*y*+1, -*x*, *z*; (xiii) *x*, *y*-1, *z*.

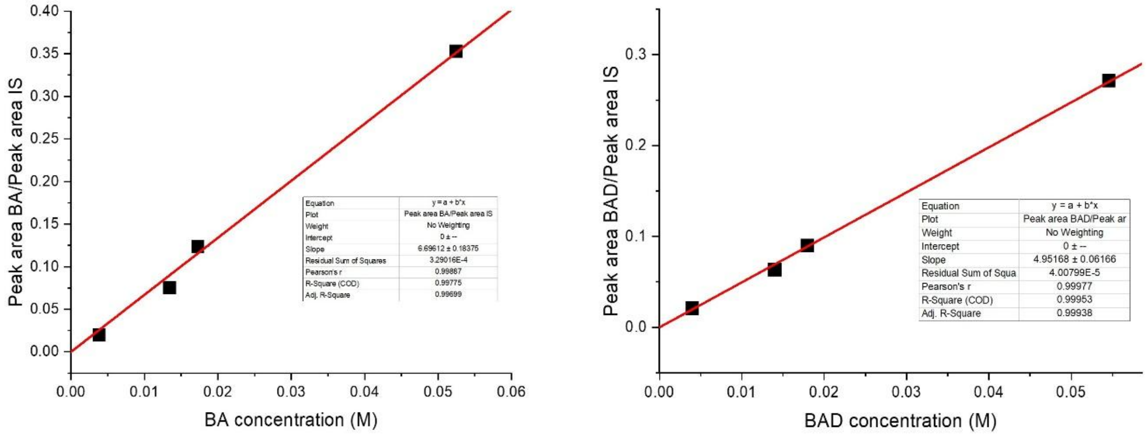

Figure 13S. GC.MS calibration curves of BA (left) and BAD (right).

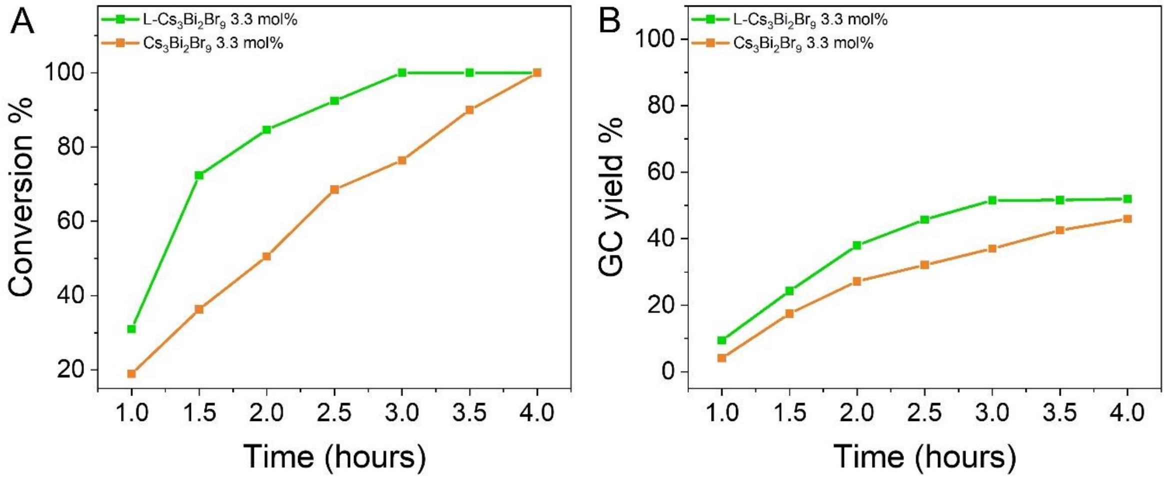

Figure 14S. Time-dependent profiles of conversion (A) and yield (B) for the two reactions promoted by the $Cs_3Bi_2Br_9$ photocatalysts.

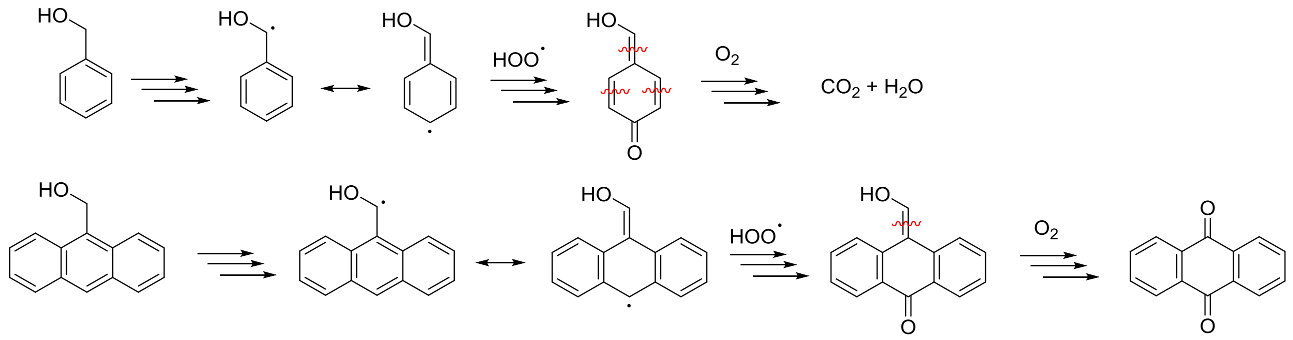

Scheme 1S. Comparison between the oxidative degradation pathways of BA (top) and anthracen-9-methanol (down).

O_2

Charge = 0 Multiplicity = 3

O	-4.96197	-7.62771	-15.53866
O	-3.64197	-7.62771	-15.53866

Zero-point correction=	0.004042 (Hartree/Particle)
Thermal correction to Energy=	0.006404
Thermal correction to Enthalpy=	0.007349
Thermal correction to Gibbs Free Energy=	0.015895
Sum of electronic and zero-point Energies=	-150.304786
Sum of electronic and thermal Energies=	-150.302424
Sum of electronic and thermal Enthalpies=	-150.301480
Sum of electronic and thermal Free Energies=	-150.324724

NUMBER OF IMAGINARY FREQUENCIES: 0

$O_2^{\bullet-}$

Charge = -1 Multiplicity = 2

O	-4.96197	-7.62771	-15.53866
O	-3.64197	-7.62771	-15.53866

NUMBER OF IMAGINARY FREQUENCIES: 0

$HO_2^{\bullet}$

Charge = 0 Multiplicity = 2

O	-4.96197	-7.62771	-15.53866
O	-3.9007	-7.15546	-15.53866
H	-5.6223	-6.93088	-15.53866

Zero-point correction=	0.014571 (Hartree/Particle)
Thermal correction to Energy=	0.017421
Thermal correction to Enthalpy=	0.018365
Thermal correction to Gibbs Free Energy=	-0.007566
Sum of electronic and zero-point Energies=	-150.882671
Sum of electronic and thermal Energies=	-150.879820
Sum of electronic and thermal Enthalpies=	-150.878876
Sum of electronic and thermal Free Energies=	-150.904807

NUMBER OF IMAGINARY FREQUENCIES: 0

IS

Charge = -3 Multiplicity = 1

Bi	-0.13902	-0.45184	-2.45053
Br	-2.27053	-0.06853	-0.56668
Br	-1.67407	0.96991	-4.82574
Br	1.73363	-1.01479	-4.96557
Br	1.26012	1.90968	-1.64253
Br	1.35165	-2.16346	-0.68417
Br	-1.58712	-2.88618	-3.99311
Bi	-0.86209	-1.47363	-6.60659
Br	0.10713	-3.88714	-7.86837
Br	-3.54086	-1.82457	-7.72241
Br	0.05484	0.23607	-8.86624
H	-0.2869	-0.81936	-11.11101
O	-0.41987	-1.23	-11.9844
C	-1.38665	-2.25315	-11.76842
H	-0.96014	-3.07133	-11.17247
H	-2.23639	-1.8723	-11.19114
C	-1.88936	-2.80233	-13.07631
C	-1.34188	-2.42361	-14.30534
C	-2.93695	-3.73589	-13.06544
C	-1.82076	-2.97111	-15.4981
H	-0.54095	-1.69443	-14.30653
C	-3.41154	-4.28267	-14.25389
H	-3.37541	-4.01835	-12.11299
C	-2.85633	-3.90411	-15.48017
H	-1.38224	-2.66543	-16.44476
H	-4.22369	-5.00315	-14.22451
H	-3.22973	-4.33052	-16.40711

Zero-point correction=	0.140356 (Hartree/Particle)
Thermal correction to Energy=	0.172986
Thermal correction to Enthalpy=	0.173930
Thermal correction to Gibbs Free Energy=	0.056980
Sum of electronic and zero-point Energies=	-476.434342
Sum of electronic and thermal Energies=	-476.401712
Sum of electronic and thermal Enthalpies=	-476.400768
Sum of electronic and thermal Free Energies=	-476.517719

NUMBER OF IMAGINARY FREQUENCIES: 0

INT1

Charge = -4 Multiplicity = 1

Bi	-1.40511	-1.34657	-2.53007
Br	-3.80408	-1.25964	-0.95635
Br	-2.66732	0.24178	-4.95976
Br	0.83959	-1.55838	-4.78076
Br	-0.2826	0.98031	-1.30008
Br	-0.0818	-3.16174	-0.73403
Br	-2.46621	-3.67178	-4.4973
Bi	-1.4612	-1.96359	-6.8261
Br	-0.17603	-4.18577	-8.15529
Br	-3.92591	-2.31996	-8.35654
Br	-0.33545	0.01193	-8.74959
H	-0.2869	-0.81936	-11.11101
O	-0.56713	-1.30304	-11.9089
C	-1.53717	-2.24003	-11.45147
H	-1.06494	-3.02168	-10.84115
C	-2.25368	-2.88055	-12.60959
C	-1.87828	-2.65551	-13.93703
C	-3.32992	-3.74328	-12.35174
C	-2.55403	-3.28476	-14.98544
H	-1.05367	-1.97989	-14.12877
C	-4.00076	-4.37167	-13.39659
H	-3.63435	-3.90586	-11.32214
C	-3.61733	-4.14699	-14.72242
H	-2.24763	-3.09857	-16.01177
H	-4.83186	-5.03525	-13.1764
H	-4.14376	-4.63686	-15.5369

Zero-point correction=	0.124168 (Hartree/Particle)
Thermal correction to Energy=	0.157032
Thermal correction to Enthalpy=	0.157977
Thermal correction to Gibbs Free Energy=	0.041055
Sum of electronic and zero-point Energies=	-475.899546
Sum of electronic and thermal Energies=	-475.866682
Sum of electronic and thermal Enthalpies=	-475.865738
Sum of electronic and thermal Free Energies=	-475.982660

NUMBER OF IMAGINARY FREQUENCIES: 0

INT2

Charge = -3 Multiplicity = 2

Bi	-1.48825	-1.46134	-3.22551
Br	-3.90573	-1.28899	-1.68758
Br	-2.67595	0.11963	-5.69722
Br	0.78121	-1.76536	-5.44053
Br	-0.324	0.85569	-2.01591
Br	-0.23649	-3.28061	-1.38294
Br	-2.5809	-3.78994	-5.17135
Bi	-1.50067	-2.14477	-7.51183
Br	-0.25422	-4.41945	-8.78803
Br	-3.95228	-2.46307	-9.07144
Br	-0.29856	-0.22902	-9.44951
H	-0.2734	-0.59366	-10.43384
O	-0.38315	-1.53336	-11.89535
C	-1.53717	-2.24003	-11.45147
H	-1.63381	-2.88278	-10.56602
C	-2.25368	-2.88055	-12.60959
C	-2.05901	-2.46585	-13.93012
C	-2.94061	-4.08655	-12.40275
C	-2.90992	-2.95681	-14.94663
H	-1.38106	-1.64124	-14.11351
C	-3.59453	-4.71944	-13.45558
H	-2.95866	-4.51313	-11.40431
C	-3.58388	-4.15884	-14.73656
H	-2.89645	-2.50824	-15.93684
H	-4.12137	-5.65184	-13.27496
H	-4.09685	-4.6534	-15.55677

Zero-point correction=	0.126901 (Hartree/Particle)
Thermal correction to Energy=	0.159385
Thermal correction to Enthalpy=	0.160329
Thermal correction to Gibbs Free Energy=	0.042386
Sum of electronic and zero-point Energies=	-475.809671
Sum of electronic and thermal Energies=	-475.777188
Sum of electronic and thermal Enthalpies=	-475.776243
Sum of electronic and thermal Free Energies=	-475.894186

NUMBER OF IMAGINARY FREQUENCIES: 0

INT3

Charge = -3 Multiplicity = 2

Bi	-1.50424	-1.46289	-3.13057
Br	-3.92172	-1.29054	-1.59264
Br	-2.69194	0.11808	-5.60228
Br	0.76522	-1.7669	-5.34559
Br	-0.33998	0.85415	-1.92097
Br	-0.25248	-3.28216	-1.288
Br	-2.59689	-3.79148	-5.07641
Bi	-1.51666	-2.14632	-7.41689
Br	-0.27021	-4.421	-8.69309
Br	-3.96827	-2.46462	-8.97649
Br	-0.31454	-0.23056	-9.35457
H	-0.27765	-0.76538	-10.79825
O	-0.36716	-1.53182	-11.99029
C	-1.52118	-2.23848	-11.54641
H	-1.22354	-2.99785	-10.85383
C	-2.23769	-2.879	-12.70453
C	-2.04302	-2.4643	-14.02506
C	-2.92463	-4.085	-12.49769
C	-2.89393	-2.95526	-15.04157
H	-1.36507	-1.6397	-14.20845
C	-3.57854	-4.7179	-13.55052
H	-2.94268	-4.51159	-11.49925
C	-3.56789	-4.1573	-14.8315
H	-2.88046	-2.5067	-16.03178
H	-4.10538	-5.6503	-13.3699
H	-4.08087	-4.65186	-15.65171
O	-2.4074	-1.32681	-10.89192
O	-3.02766	-0.59037	-11.79489

Zero-point correction=	0.137013 (Hartree/Particle)
Thermal correction to Energy=	0.171304
Thermal correction to Enthalpy=	0.172248
Thermal correction to Gibbs Free Energy=	0.051411
Sum of electronic and zero-point Energies=	-626.155216
Sum of electronic and thermal Energies=	-626.120925
Sum of electronic and thermal Enthalpies=	-626.119981
Sum of electronic and thermal Free Energies=	-626.240818

NUMBER OF IMAGINARY FREQUENCIES: 0

TS1

Charge = -3 Multiplicity = 2

Bi	-1.45334	-2.16659	-3.20743
Br	-3.56047	-2.91485	-1.49576
Br	-3.25654	-0.35957	-5.04802
Br	0.47282	-1.3744	-5.56295
Br	-0.63737	-0.03831	-1.54845
Br	0.36302	-3.98397	-2.06666
Br	-2.30337	-4.12545	-5.48466
Bi	-1.90879	-1.7609	-7.40773
Br	-0.42773	-3.31239	-9.29868
Br	-4.3556	-2.24895	-8.84741
Br	-1.45694	0.60668	-8.93031
H	-1.61397	0.27346	-12.75866
O	-0.96679	-0.69556	-12.56283
C	-1.82611	-1.44687	-11.99565
H	-2.05523	-1.32769	-10.93668
C	-2.21047	-2.72942	-12.64555
C	-2.01908	-2.88479	-14.0405
C	-2.75223	-3.7724	-11.85898
C	-2.38806	-4.0781	-14.64922
H	-1.58229	-2.06191	-14.6138
C	-3.10732	-4.96263	-12.47623
H	-2.89378	-3.63311	-10.78292
C	-2.93064	-5.11815	-13.86932
H	-2.23737	-4.19409	-15.73963
H	-3.5267	-5.77261	-11.86628
H	-3.20085	-6.05402	-14.33851
O	-3.39331	-0.28046	-12.50901
O	-2.73798	0.72318	-12.9433

Zero-point correction=	0.130056 (Hartree/Particle)
Thermal correction to Energy=	0.164127
Thermal correction to Enthalpy=	0.165071
Thermal correction to Gibbs Free Energy=	0.044836
Sum of electronic and zero-point Energies=	-626.127809
Sum of electronic and thermal Energies=	-626.093738
Sum of electronic and thermal Enthalpies=	-626.092794
Sum of electronic and thermal Free Energies=	-626.213029

NUMBER OF IMAGINARY FREQUENCIES: 1 (-901.64 cm^{-1})

$Bi_2Br_9^{3-}$

Charge = -3 Multiplicity = 1

Bi	-1.07248	-0.42815	-2.5441
Br	-3.48996	-0.2558	-1.00617
Br	-2.26018	1.15282	-5.01581
Br	1.19698	-0.73217	-4.75912
Br	0.09178	1.88888	-1.3345
Br	0.17928	-2.24742	-0.70153
Br	-2.16513	-2.75675	-4.48994
Bi	-1.0849	-1.11159	-6.83042
Br	0.16155	-3.38627	-8.10662
Br	-3.5365	-1.42989	-8.39003
Br	0.11722	0.80417	-8.7681

Zero-point correction=	0.005022 (Hartree/Particle)
Thermal correction to Energy=	0.028774
Thermal correction to Enthalpy=	0.029718
Thermal correction to Gibbs Free Energy=	-0.065246
Sum of electronic and zero-point Energies=	-129.825201
Sum of electronic and thermal Energies=	-129.801449
Sum of electronic and thermal Enthalpies=	-129.800505
Sum of electronic and thermal Free Energies=	-129.895469

NUMBER OF IMAGINARY FREQUENCIES: 0

BAD (FS-free)

Charge = 0 Multiplicity = 1

O	-0.79893	-2.56655	-12.57676
C	-1.95294	-3.27322	-12.13288
H	-2.04958	-3.91596	-11.24743
C	-2.66945	-3.91373	-13.291
C	-2.47478	-3.49903	-14.61152
C	-3.35639	-5.11973	-13.08416
C	-3.32569	-3.99	-15.62804
H	-1.79684	-2.67443	-14.79492
C	-4.0103	-5.75263	-14.13699
H	-3.37444	-5.54632	-12.08571
C	-3.99965	-5.19203	-15.41797
H	-3.31223	-3.54143	-16.61825
H	-4.53714	-6.68503	-13.95637
H	-4.51263	-5.68659	-16.23818

Zero-point correction=	0.110366 (Hartree/Particle)
Thermal correction to Energy=	0.116702
Thermal correction to Enthalpy=	0.117646
Thermal correction to Gibbs Free Energy=	0.079728
Sum of electronic and zero-point Energies=	-345.412810
Sum of electronic and thermal Energies=	-345.406474
Sum of electronic and thermal Enthalpies=	-345.405530
Sum of electronic and thermal Free Energies=	-345.443448

NUMBER OF IMAGINARY FREQUENCIES: 0

IS-cat

Charge = -2 Multiplicity = 2

Bi	-0.13902	-0.45184	-2.45053
Br	-2.27053	-0.06853	-0.56668
Br	-1.67407	0.96991	-4.82574
Br	1.73363	-1.01479	-4.96557
Br	1.26012	1.90968	-1.64253
Br	1.35165	-2.16346	-0.68417
Br	-1.58712	-2.88618	-3.99311
Bi	-0.86209	-1.47363	-6.60659
Br	0.10713	-3.88714	-7.86837
Br	-3.54086	-1.82457	-7.72241
Br	0.05484	0.23607	-8.86624
H	-0.2869	-0.81936	-11.11101
O	-0.41987	-1.23	-11.9844
C	-1.38665	-2.25315	-11.76842
H	-0.96014	-3.07133	-11.17247
H	-2.23639	-1.8723	-11.19114
C	-1.88936	-2.80233	-13.07631
C	-1.34188	-2.42361	-14.30534
C	-2.93695	-3.73589	-13.06544
C	-1.82076	-2.97111	-15.4981
H	-0.54095	-1.69443	-14.30653
C	-3.41154	-4.28267	-14.25389
H	-3.37541	-4.01835	-12.11299
C	-2.85633	-3.90411	-15.48017
H	-1.38224	-2.66543	-16.44476
H	-4.22369	-5.00315	-14.22451
H	-3.22973	-4.33052	-16.40711

Zero-point correction=	0.138407 (Hartree/Particle)
Thermal correction to Energy=	0.171194
Thermal correction to Enthalpy=	0.172139
Thermal correction to Gibbs Free Energy=	0.055473
Sum of electronic and zero-point Energies=	-476.192373
Sum of electronic and thermal Energies=	-476.159586
Sum of electronic and thermal Enthalpies=	-476.158641
Sum of electronic and thermal Free Energies=	-476.275307

NUMBER OF IMAGINARY FREQUENCIES: 0

TS2

Charge = -2 Multiplicity = 2

Br	-1.04337	0.22472	-9.85601
Br	1.82072	-2.77625	-10.25538
H	-0.00995	-0.00809	-12.62811
O	0.9302	-0.04787	-13.06867
C	1.80457	-0.24823	-12.05245
H	1.53673	0.23968	-11.10781
H	1.69937	-1.6204	-11.55239
C	3.19992	-0.21493	-12.51261
C	3.46695	-0.35975	-13.88992
C	4.28654	-0.13201	-11.61928
C	4.77307	-0.38333	-14.36221
H	2.63412	-0.44748	-14.58034
C	5.59378	-0.15723	-12.0984
H	4.11014	-0.04857	-10.53919
C	5.84494	-0.28521	-13.47026
H	4.95927	-0.4952	-15.42991
H	6.42696	-0.08691	-11.39509
H	6.85995	-0.31041	-13.83866
Bi	-0.2551	-1.57463	-7.83699
Br	-1.67109	-0.18145	-5.94753
Br	2.21176	0.02706	-6.83616
Br	0.6293	-3.38531	-5.37982
Bi	1.16318	-0.74381	-3.77929
Br	-0.19741	-1.80929	-1.50284
Br	1.3392	1.87882	-2.83318
Br	3.76928	-1.52091	-2.94809
Br	-2.46955	-3.04136	-8.83378

Zero-point correction=	0.131675 (Hartree/Particle)
Thermal correction to Energy=	0.163057
Thermal correction to Enthalpy=	0.164002
Thermal correction to Gibbs Free Energy=	0.049029
Sum of electronic and zero-point Energies=	-476.171436
Sum of electronic and thermal Energies=	-476.140054
Sum of electronic and thermal Enthalpies=	-476.139110
Sum of electronic and thermal Free Energies=	-476.254082

NUMBER OF IMAGINARY FREQUENCIES: 1 (-669.32 cm^{-1})

INT4

Charge = -2 Multiplicity = 2

Bi	-1.70642	-2.76223	-4.5817
Br	-4.3609	-2.1765	-4.42415
Br	-0.86822	0.18731	-5.64696
Br	1.60872	-2.85951	-5.73067
Br	-1.1512	-2.18705	-1.93532
Br	-1.99239	-5.46501	-4.18043
Br	-1.80128	-3.01246	-7.70686
Bi	0.69293	-1.16475	-7.71697
Br	1.87327	-2.67899	-9.93396
Br	-1.05064	0.38564	-10.15224
Br	2.75516	0.67208	-7.66319
H	1.15815	-3.11776	-12.11001
O	0.49932	-3.25567	-12.82154
C	-0.72628	-3.17039	-12.25075
H	-0.79564	-3.56814	-11.23057
H	-1.3286	-1.28432	-10.81703
C	-1.85263	-3.51082	-13.14165
C	-1.66234	-3.69664	-14.51321
C	-3.13629	-3.6156	-12.59297
C	-2.74691	-3.9937	-15.32872
H	-0.66179	-3.61266	-14.91944
C	-4.21443	-3.9078	-13.41641
H	-3.27363	-3.46687	-11.52594
C	-4.02458	-4.09902	-14.78342
H	-2.59657	-4.14387	-16.39219
H	-5.20618	-3.98754	-12.9869
H	-4.87065	-4.32788	-15.42203

Zero-point correction=	0.136393 (Hartree/Particle)
Thermal correction to Energy=	0.168597
Thermal correction to Enthalpy=	0.169541
Thermal correction to Gibbs Free Energy=	0.053013
Sum of electronic and zero-point Energies=	-476.198406
Sum of electronic and thermal Energies=	-476.166202
Sum of electronic and thermal Enthalpies=	-476.165258
Sum of electronic and thermal Free Energies=	-476.281786

NUMBER OF IMAGINARY FREQUENCIES: 0

TS3

Charge = -2 Multiplicity = 2

Bi	-0.85787	-2.24115	-4.77445
Br	-3.46446	-1.59094	-4.28104
Br	-0.28375	0.41391	-6.45947
Br	2.20711	-2.69618	-6.09154
Br	0.13397	-1.24478	-2.41883
Br	-1.06754	-4.85481	-3.91054
Br	-1.25758	-3.07526	-7.90601
Bi	1.0816	-1.42707	-8.37492
Br	2.00431	-3.16899	-10.43725
Br	-0.37291	0.11908	-10.61674
Br	3.27102	0.4785	-9.12635
H	3.06079	1.30035	-10.83089
O	2.87116	1.21259	-12.13392
C	2.2668	0.0191	-12.24483
H	2.61724	-0.73918	-11.48159
H	1.09102	-0.03404	-11.85657
C	2.15698	-0.52909	-13.63545
C	2.43224	0.27656	-14.74274
C	1.67545	-1.83745	-13.80942
C	2.23077	-0.23641	-16.02799
H	2.80256	1.27877	-14.58904
C	1.47431	-2.33412	-15.08669
H	1.46355	-2.44327	-12.92452
C	1.74713	-1.53491	-16.20264
H	2.45534	0.37867	-16.89107
H	1.11211	-3.34894	-15.21905
H	1.58013	-1.92189	-17.19795

Zero-point correction=	0.129849 (Hartree/Particle)
Thermal correction to Energy=	0.161360
Thermal correction to Enthalpy=	0.162304
Thermal correction to Gibbs Free Energy=	0.048461
Sum of electronic and zero-point Energies=	-476.156169
Sum of electronic and thermal Energies=	-476.124657
Sum of electronic and thermal Enthalpies=	-476.123713
Sum of electronic and thermal Free Energies=	-476.237557

NUMBER OF IMAGINARY FREQUENCIES: 1 (-1387.85 cm^{-1})

$H_2Bi_2Br_9^{3-}$

Charge = -3 Multiplicity = 1

Bi	-1.07248	-0.42815	-2.5441
Br	-3.48996	-0.2558	-1.00617
Br	-2.26018	1.15282	-5.01581
Br	1.19698	-0.73217	-4.75912
Br	0.09178	1.88888	-1.3345
Br	0.17928	-2.24742	-0.70153
Br	-2.16513	-2.75675	-4.48994
Bi	-1.0849	-1.11159	-6.83042
Br	0.16155	-3.38627	-8.10662
Br	-3.5365	-1.42989	-8.39003
Br	0.11722	0.80417	-8.7681
H	0.14238	0.43952	-9.75243
H	0.78245	-4.51937	-8.74234

Zero-point correction=	0.018333 (Hartree/Particle)
Thermal correction to Energy=	0.044563
Thermal correction to Enthalpy=	0.045508
Thermal correction to Gibbs Free Energy=	-0.058531
Sum of electronic and zero-point Energies=	-130.881363
Sum of electronic and thermal Energies=	-130.855133
Sum of electronic and thermal Enthalpies=	-130.854189
Sum of electronic and thermal Free Energies=	-130.958227

NUMBER OF IMAGINARY FREQUENCIES: 0

IS-hv

Charge = -2 Multiplicity = 1

Bi	-0.7383	-1.14632	-0.91268
Bi	-0.49096	-3.54468	2.4967
Br	-1.19233	-2.18106	-3.41528
Br	-2.81345	-3.21773	0.72853
Br	-0.10043	-0.6548	2.26994
Br	1.12382	-3.57206	-0.05523
Br	1.44556	0.34593	-1.63313
Br	-1.38005	-3.20147	5.14906
Br	0.34748	-6.2705	3.02411
O	1.77978	-3.42746	3.29024
H	2.07171	-4.3479	3.37126
C	2.82924	-2.53051	2.91369
H	3.67481	-3.10467	2.52844
H	2.45145	-1.91069	2.09717
C	3.22903	-1.67452	4.08634
C	4.52798	-1.18077	4.18391
C	2.29217	-1.341	5.06415
C	4.8948	-0.35796	5.24477
H	5.2564	-1.43838	3.42075
C	2.66446	-0.53062	6.13023
H	1.28047	-1.72586	4.99763
C	3.96168	-0.03329	6.22386
H	5.90832	0.02299	5.30834
H	1.92886	-0.28093	6.88633
H	4.24363	0.60265	7.05595
Br	-2.54744	0.927	-1.14821

Zero-point correction=	0.140690 (Hartree/Particle)
Thermal correction to Energy=	0.170484
Thermal correction to Enthalpy=	0.171429
Thermal correction to Gibbs Free Energy=	0.062589
Sum of electronic and zero-point Energies=	-463.117318
Sum of electronic and thermal Energies=	-463.087523
Sum of electronic and thermal Enthalpies=	-463.086579
Sum of electronic and thermal Free Energies=	-463.195419

NUMBER OF IMAGINARY FREQUENCIES: 0

INT5

Charge = -3 Multiplicity = 1

Bi	-1.22585	-1.83176	-1.85512
Bi	-0.34707	-2.70185	2.18101
Br	-2.25803	-3.70173	-3.64599
Br	-2.95812	-2.91123	0.56458
Br	0.00241	-0.2032	0.66984
Br	0.71394	-3.83223	-0.54646
Br	0.66931	-0.86776	-3.6747
Br	-1.4495	-1.53447	4.44936
Br	0.19246	-5.22765	3.49061
O	1.81971	-2.48148	2.92806
H	2.15982	-3.33656	3.23553
C	2.70642	-1.40359	3.20511
H	2.9346	-0.81456	2.3324
C	3.21801	-1.25527	4.46611
C	4.21958	-0.25211	4.76326
C	2.84655	-2.0922	5.58701
C	4.7977	-0.14572	6.00611
H	4.51723	0.4256	3.96823
C	3.45477	-1.95507	6.81777
H	2.04313	-2.81144	5.46433
C	4.45163	-0.99854	7.07342
H	5.54796	0.62851	6.16299
H	3.12807	-2.60986	7.6236
H	4.90957	-0.90046	8.051
Br	-3.12041	0.14467	-2.53961

Zero-point correction=	0.123890 (Hartree/Particle)
Thermal correction to Energy=	0.153516
Thermal correction to Enthalpy=	0.154460
Thermal correction to Gibbs Free Energy=	0.045978
Sum of electronic and zero-point Energies=	-462.599447
Sum of electronic and thermal Energies=	-462.569822
Sum of electronic and thermal Enthalpies=	-462.568877
Sum of electronic and thermal Free Energies=	-462.677360

NUMBER OF IMAGINARY FREQUENCIES: 0

INT6

Charge = -2 Multiplicity = 2

Bi	-0.01305	-2.64941	-2.59795
Bi	-0.197	-2.58459	1.59161
Br	-1.39785	-4.44576	-4.13283
Br	-2.43768	-2.27829	-0.17767
Br	1.36436	-0.88466	-0.14592
Br	0.68594	-4.72736	-0.33296
Br	2.38452	-3.01184	-3.84863
Br	-0.68944	-0.48718	3.38479
Br	-0.26777	-4.5056	3.85837
O	2.04417	-2.62301	2.7767
H	1.85223	-3.22682	3.51909
C	2.77253	-1.53499	3.12745
H	2.71509	-0.73758	2.40046
C	3.47257	-1.44638	4.3445
C	4.36987	-0.36894	4.55052
C	3.31758	-2.3976	5.38423
C	5.09953	-0.26989	5.7183
H	4.47516	0.37877	3.77173
C	4.06689	-2.28712	6.54191
H	2.57706	-3.18759	5.30149
C	4.96558	-1.23401	6.72279
H	5.78119	0.56289	5.85416
H	3.9283	-3.01986	7.32902
H	5.54042	-1.15508	7.63807
Br	-0.73192	-0.43119	-4.08357

Zero-point correction=	0.126441 (Hartree/Particle)
Thermal correction to Energy=	0.156372
Thermal correction to Enthalpy=	0.157316
Thermal correction to Gibbs Free Energy=	0.046557
Sum of electronic and zero-point Energies=	-462.482849
Sum of electronic and thermal Energies=	-462.452918
Sum of electronic and thermal Enthalpies=	-462.451973
Sum of electronic and thermal Free Energies=	-462.562733

NUMBER OF IMAGINARY FREQUENCIES: 0

INT7

Charge = -2 Multiplicity = 2

Bi	-1.50087	-2.19701	-2.031
Bi	-0.26044	-2.39163	1.97183
Br	-2.17367	-4.43407	-3.47609
Br	-2.74556	-3.31655	1.0575
Br	-0.55175	-0.06898	0.25508
Br	0.87477	-3.62435	-0.53213
Br	0.13056	-1.12602	-3.92998
Br	-1.08073	-1.09075	4.33651
Br	0.86956	-4.58217	3.62887
O	2.30189	-1.96493	2.56545
H	2.38833	-2.78002	3.09686
C	2.98789	-0.89848	3.11468
C	3.40304	-1.11051	4.54457
C	4.68043	-0.74464	4.95632
C	2.50014	-1.66278	5.45393
C	5.06724	-0.9338	6.27906
H	5.36826	-0.30791	4.24005
C	2.89089	-1.84807	6.77383
H	1.49632	-1.93192	5.14011
C	4.1712	-1.48917	7.18697
H	6.06353	-0.64978	6.59807
H	2.18817	-2.27864	7.47715
H	4.47008	-1.64083	8.21836
Br	-3.74559	-0.75665	-2.7605
H	3.82736	-0.59694	2.48361
O	2.03432	0.20674	3.03332
O	2.63588	1.34044	3.17207

Zero-point correction=	0.136700 (Hartree/Particle)
Thermal correction to Energy=	0.168551
Thermal correction to Enthalpy=	0.169496
Thermal correction to Gibbs Free Energy=	0.054048
Sum of electronic and zero-point Energies=	-612.818970
Sum of electronic and thermal Energies=	-612.787118
Sum of electronic and thermal Enthalpies=	-612.786174
Sum of electronic and thermal Free Energies=	-612.901621

NUMBER OF IMAGINARY FREQUENCIES: 0

TS4

Charge = -2 Multiplicity = 2

O	-1.01055	-2.65565	-12.99656
C	-1.61944	-3.79233	-12.49718
H	-2.33072	-4.28106	-13.15202
C	-1.08169	-4.40328	-11.3044
C	-0.12575	-3.72221	-10.53507
C	-1.56993	-5.65028	-10.88628
C	0.3271	-4.28302	-9.3495
H	0.2612	-2.7571	-10.85387
C	-1.11283	-6.19863	-9.70246
H	-2.2836	-6.17773	-11.50992
C	-0.16567	-5.51338	-8.93624
H	1.09728	-3.77804	-8.77854
H	-1.47227	-7.16834	-9.37758
H	0.21184	-5.95998	-8.02282
H	-1.37432	-1.84511	-12.36142
O	-3.04797	-2.45326	-11.46097
O	-2.33447	-1.38243	-11.57303
Bi	1.35338	-3.47419	-13.85175
Br	3.84618	-3.85241	-15.04898
Br	2.37644	-1.34115	-12.09466
Br	2.68062	-5.34039	-11.69837
Br	0.53269	-1.82544	-15.9151
Br	-0.26618	-5.71178	-14.70334
Bi	4.97602	-3.30056	-11.78942
Br	6.99273	-5.15684	-11.93836
Br	4.96787	-3.08305	-9.06018
Br	6.67992	-1.15403	-12.26993

Zero-point correction=	0.129895 (Hartree/Particle)
Thermal correction to Energy=	0.161501
Thermal correction to Enthalpy=	0.162445
Thermal correction to Gibbs Free Energy=	0.047903
Sum of electronic and zero-point Energies=	-612.794073
Sum of electronic and thermal Energies=	-612.762467
Sum of electronic and thermal Enthalpies=	-612.761523
Sum of electronic and thermal Free Energies=	-612.876065

NUMBER OF IMAGINARY FREQUENCIES: 1 (-417.08 cm^{-1})

$Bi_2Br_8^{2-}$

Charge = -2 Multiplicity = 1

Bi	-0.71096	-1.58898	-0.9364
Bi	-0.16963	-3.41798	2.07258
Br	-0.28978	-2.54541	-3.38251
Br	-1.83964	-3.78344	0.01467
Br	-1.05552	-0.94632	1.60778
Br	1.4796	-2.64334	0.10541
Br	0.46983	0.6739	-1.68521
Br	-1.80555	-4.18456	4.02505
Br	0.78373	-5.899	2.18296
Br	-2.97254	-0.52267	-1.84406

Zero-point correction=	0.004752 (Hartree/Particle)
Thermal correction to Energy=	0.025954
Thermal correction to Enthalpy=	0.026898
Thermal correction to Gibbs Free Energy=	-0.060819
Sum of electronic and zero-point Energies=	-116.490241
Sum of electronic and thermal Energies=	-116.469039
Sum of electronic and thermal Enthalpies=	-116.468095
Sum of electronic and thermal Free Energies=	-116.555812

NUMBER OF IMAGINARY FREQUENCIES: 0

IS-free (BA)

Charge = 0 Multiplicity = 1

O	-0.79893	-2.56655	-12.57676
C	-1.95294	-3.27322	-12.13288
H	-1.6553	-4.03258	-11.44029
C	-2.66945	-3.91373	-13.291
C	-2.47478	-3.49903	-14.61152
C	-3.35639	-5.11973	-13.08416
C	-3.32569	-3.99	-15.62804
H	-1.79684	-2.67443	-14.79492
C	-4.0103	-5.75263	-14.13699
H	-3.37444	-5.54632	-12.08571
C	-3.99965	-5.19203	-15.41797
H	-3.31223	-3.54143	-16.61825
H	-4.53714	-6.68503	-13.95637
H	-4.51263	-5.68659	-16.23818
H	-0.00926	-3.02883	-12.28638
H	-2.61606	-2.59105	-11.64316

Zero-point correction=	0.134167 (Hartree/Particle)
Thermal correction to Energy=	0.141203
Thermal correction to Enthalpy=	0.142147
Thermal correction to Gibbs Free Energy=	0.102098
Sum of electronic and zero-point Energies=	-346.592860
Sum of electronic and thermal Energies=	-346.585824
Sum of electronic and thermal Enthalpies=	-346.584879
Sum of electronic and thermal Free Energies=	-346.624928

NUMBER OF IMAGINARY FREQUENCIES: 0

INT8

Charge = -1 Multiplicity = 1

O	-0.79893	-2.56655	-12.57676
C	-1.95294	-3.27322	-12.13288
H	-1.6553	-4.03258	-11.44029
C	-2.66945	-3.91373	-13.291
C	-2.47478	-3.49903	-14.61152
C	-3.35639	-5.11973	-13.08416
C	-3.32569	-3.99	-15.62804
H	-1.79684	-2.67443	-14.79492
C	-4.0103	-5.75263	-14.13699
H	-3.37444	-5.54632	-12.08571
C	-3.99965	-5.19203	-15.41797
H	-3.31223	-3.54143	-16.61825
H	-4.53714	-6.68503	-13.95637
H	-4.51263	-5.68659	-16.23818
H	-0.00926	-3.02883	-12.28638

Zero-point correction=	0.117338 (Hartree/Particle)
Thermal correction to Energy=	0.124742
Thermal correction to Enthalpy=	0.125687
Thermal correction to Gibbs Free Energy=	0.085902
Sum of electronic and zero-point Energies=	-346.068379
Sum of electronic and thermal Energies=	-346.060974
Sum of electronic and thermal Enthalpies=	-346.060030
Sum of electronic and thermal Free Energies=	-346.099815

NUMBER OF IMAGINARY FREQUENCIES: 0

INT9

Charge = 0 Multiplicity = 2

O	-0.79893	-2.56655	-12.57676
C	-1.95294	-3.27322	-12.13288
H	-1.6553	-4.03258	-11.44029
C	-2.66945	-3.91373	-13.291
C	-2.47478	-3.49903	-14.61152
C	-3.35639	-5.11973	-13.08416
C	-3.32569	-3.99	-15.62804
H	-1.79684	-2.67443	-14.79492
C	-4.0103	-5.75263	-14.13699
H	-3.37444	-5.54632	-12.08571
C	-3.99965	-5.19203	-15.41797
H	-3.31223	-3.54143	-16.61825
H	-4.53714	-6.68503	-13.95637
H	-4.51263	-5.68659	-16.23818
H	-0.00926	-3.02883	-12.28638

Zero-point correction=	0.120234 (Hartree/Particle)
Thermal correction to Energy=	0.127107
Thermal correction to Enthalpy=	0.128051
Thermal correction to Gibbs Free Energy=	0.088773
Sum of electronic and zero-point Energies=	-345.966422
Sum of electronic and thermal Energies=	-345.959549
Sum of electronic and thermal Enthalpies=	-345.958605
Sum of electronic and thermal Free Energies=	-345.997883

NUMBER OF IMAGINARY FREQUENCIES: 0

INT10

Charge = 0 Multiplicity = 2

O	-0.79893	-2.56655	-12.57676
C	-1.95294	-3.27322	-12.13288
H	-1.6553	-4.03258	-11.44029
C	-2.66945	-3.91373	-13.291
C	-2.47478	-3.49903	-14.61152
C	-3.35639	-5.11973	-13.08416
C	-3.32569	-3.99	-15.62804
H	-1.79684	-2.67443	-14.79492
C	-4.0103	-5.75263	-14.13699
H	-3.37444	-5.54632	-12.08571
C	-3.99965	-5.19203	-15.41797
H	-3.31223	-3.54143	-16.61825
H	-4.53714	-6.68503	-13.95637
H	-4.51263	-5.68659	-16.23818
H	-0.00926	-3.02883	-12.28638
O	-2.83916	-2.36154	-11.47839
O	-2.46402	-2.20752	-10.22223

Zero-point correction=	0.130344 (Hartree/Particle)
Thermal correction to Energy=	0.139041
Thermal correction to Enthalpy=	0.139986
Thermal correction to Gibbs Free Energy=	0.094881
Sum of electronic and zero-point Energies=	-496.309966
Sum of electronic and thermal Energies=	-496.301268
Sum of electronic and thermal Enthalpies=	-496.300324
Sum of electronic and thermal Free Energies=	-496.345428

NUMBER OF IMAGINARY FREQUENCIES: 0

TS5

Charge = 0 Multiplicity = 2

O	-0.65935	-2.93242	-12.55381
C	-1.85947	-3.4667	-12.15512
H	-1.76237	-4.14643	-11.30494
C	-2.60189	-4.07334	-13.30468
C	-2.60127	-3.43761	-14.54595
C	-3.31802	-5.25223	-13.121
C	-3.31634	-3.98797	-15.60257
H	-2.03842	-2.52111	-14.67918
C	-4.03347	-5.80198	-14.18125
H	-3.31357	-5.74289	-12.15371
C	-4.03195	-5.17043	-15.42061
H	-3.31556	-3.49691	-16.56821
H	-4.58612	-6.7227	-14.03924
H	-4.58667	-5.59933	-16.2468
H	-0.42869	-2.43396	-11.48689
O	-2.8034	-2.28026	-11.6134
O	-1.8747	-1.85161	-10.8458

Zero-point correction=	0.124790 (Hartree/Particle)
Thermal correction to Energy=	0.133159
Thermal correction to Enthalpy=	0.134103
Thermal correction to Gibbs Free Energy=	0.089661
Sum of electronic and zero-point Energies=	-496.291300
Sum of electronic and thermal Energies=	-496.282932
Sum of electronic and thermal Enthalpies=	-496.281988
Sum of electronic and thermal Free Energies=	-496.326430

NUMBER OF IMAGINARY FREQUENCIES: 1 (–907.75 cm^{-1})

REFERENCES

S1. N. Fairley, V. Fernandez, M. Richard-Plouet, C. Guillot-Deudon, J. Walton, E. Smith, D. Flahaut, M. Greiner, M. Biesinger, S. Tougaard, D. Morgan, J. Baltrusaitis, *Appl. Surf. Sci. Adv.* **2021**, *5*, 100112.

S2. J.H. Scofield, *J. Electron Spectr. Relat. Phenom.* **1976**, *8*, 129–137.

S3. H. Shinotsuka, S. Tanuma, C. J. Powell, D. R. Penn, *Surf. Interface Anal.* **2015**, *47*, 871–888.

S4. A. Altomare, C. Cuocci, C. Giacovazzo, A. Moliterni, R. Rizzi, N. Corriero, A. Falcicchio, *J. Appl. Cryst.* **2013**, *46*, 1231–1235.

S5. A. Altomare, G. Campi, C. Cuocci, L. Eriksson, C. Giacovazzo, A. Moliterni, R. Rizzi, P. E. Werner, *J. Appl. Cryst.* **2009**, *42*, 768–775

S6. P. M. de Wolff, A Simplified Criterion for the Reliability of a Powder Pattern Indexing. *J. Appl. Cryst.* **1968**, *1*, 108–113.

S7. A. Altomare, R. Caliandro, M. Camalli, C. Cuocci, I. Da Silva, C. Giacovazzo, A. G. G. Moliterni, R. Spagna, *J. Appl. Cryst.* **2004**, *37*, 957–966.

S8. A. Altomare, M. Camalli, C. Cuocci, C. Giacovazzo, A. G. G. Moliterni, R. Rizzi, *J. Appl. Cryst.* **2007**, *40*, 743–748.

S9. A. Le Bail, H. Duroy. J.L Fourquet, *Mater. Res. Bull.* **1988**, *23*, 447–452.

S10. C. Giacovazzo, Phasing in Crystallography: A Modern Perspective. **2013**, International Union of Crystallography/Oxford University Press.

S11. H. M. Rietveld, *J. Appl. Cryst.* **1969**, *2*, 65–71.

S12. R. A. Young, Introduction to the Rietveld method. In *The Rietveld Method*; Young, R.A., Ed.; Oxford University Press: New York, NY, USA, 1996; p. 22.

S13. C. F. Macrae, I. Sovago, S. J. Cottrell, P. T. Galek, P. McCabe, E. Pidcock, M. Platings, G. P. Shields, J. S. Stevens, M. Towler, P. A. Wood *J. Appl. Cryst*. **2020**, *53*, 226–235.

S14. https://checkcif.iucr.org (IUCr checkCIF/PLATON service)

S15. S. W. Westrip, *J. Appl. Cryst.* **2010**, *43*, 920–925.
